# Next‐Generation Brain Health Monitoring Enabled by Integrated Wearable AI Infrastructure

**DOI:** 10.1002/smmd.70053

**Published:** 2026-10-26

**Authors:** Muhammad Hasan Masrur, Bangul Khan, Rana Talha Khalid, Sumaira, Saad Abdullah

**Affiliations:** ^1^ James Watt School of Engineering University of Glasgow Glasgow UK; ^2^ Department of Biomedical Engineering College of Biomedicine City University of Hong Kong Kowloon Hong Kong; ^3^ Hong Kong Centre of Cerebro‐Cardiovascular Health Engineering (COCHE) Shatin Hong Kong; ^4^ Department of Biomedical Engineering Riphah International University Lahore Pakistan; ^5^ Mehran University of Engineering and Technology Jamshoro Sindh Pakistan; ^6^ Department of Computer Science and Engineering Mälardalen University Västerås Sweden

**Keywords:** AI‐enhanced wearable sensors, bio‐signal monitoring, and remote patient monitoring, digital biomarkers, edge AI, multimodal fusion

## Abstract

Neurodegenerative diseases are difficult to detect during their pre‐symptomatic stages, when intervention is most effective. Advances in wearable biosensors, artificial intelligence (AI), and neuroscience now enable continuous monitoring of brain‐related physiological signals in real‐world settings. However, progress in sensing technologies, signal analytics, and machine learning remains fragmented, limiting the biological specificity, robustness, and clinical translation of many digital biomarkers. This review presents an integrated framework that links molecular pathology and neural circuit dysfunction to sensor‐detectable digital phenotypes across sleep, autonomic regulation, gait, speech, and ocular dynamics. We evaluate emerging wearable platforms, including bioelectric, optical, and neuromotor sensors, and discuss critical design considerations such as signal quality, user‐centered form factors, and ultra‐low‐power edge AI systems. We further examine how self‐supervised learning, federated AI, mechanistic modeling, and fairness‐aware validation can improve biomarker reliability, interpretability, and generalizability. To facilitate clinical deployment, we introduce the Personalized Brain‐Health Operating System (pBH‐OS), a privacy‐preserving reference architecture for scalable digital phenotyping. The framework maps conceptual layers to existing technologies while identifying key validation gaps. We also address practical challenges, including adherence, device dropout, informative missingness, regulatory compliance, and accountability. Together, these advances provide a pathway toward biologically grounded and regulation‐ready systems capable of identifying neurodegenerative risk years before clinical symptom onset.

## Highlights

- Links molecular pathology to neural dysfunction and wearable‐detectable digital phenotypes.
- Summarizes multimodal wearable platforms and key sensing design principles.
- Evaluates self‐supervised learning, federated models, and edge‐AI for robust biomarkers.
- Identifies significant gaps in biological validity, generalization, and regulatory readiness.
- Introduces a privacy‐preserving, scalable Brain‐Health Operating System with defined acceptance criteria and a mapping to deployed systems.

## Graphical abstract

This review presents an integrated framework for next‐generation brain‐health monitoring that connects molecular pathology to digital biomarkers via multimodal wearable sensing, signal processing, and AI‐driven analytics. It highlights advances in sensor design, data quality, edge computing, artificial intelligence, digital twins, and clinical translation. The framework culminates in the Personalized Brain‐Health Operating System (pBH‐OS): a unified, privacy‐preserving, fairness‐aware, and sustainability‐oriented infrastructure for early, scalable, and equitable detection of neurodegenerative disease at the population level.

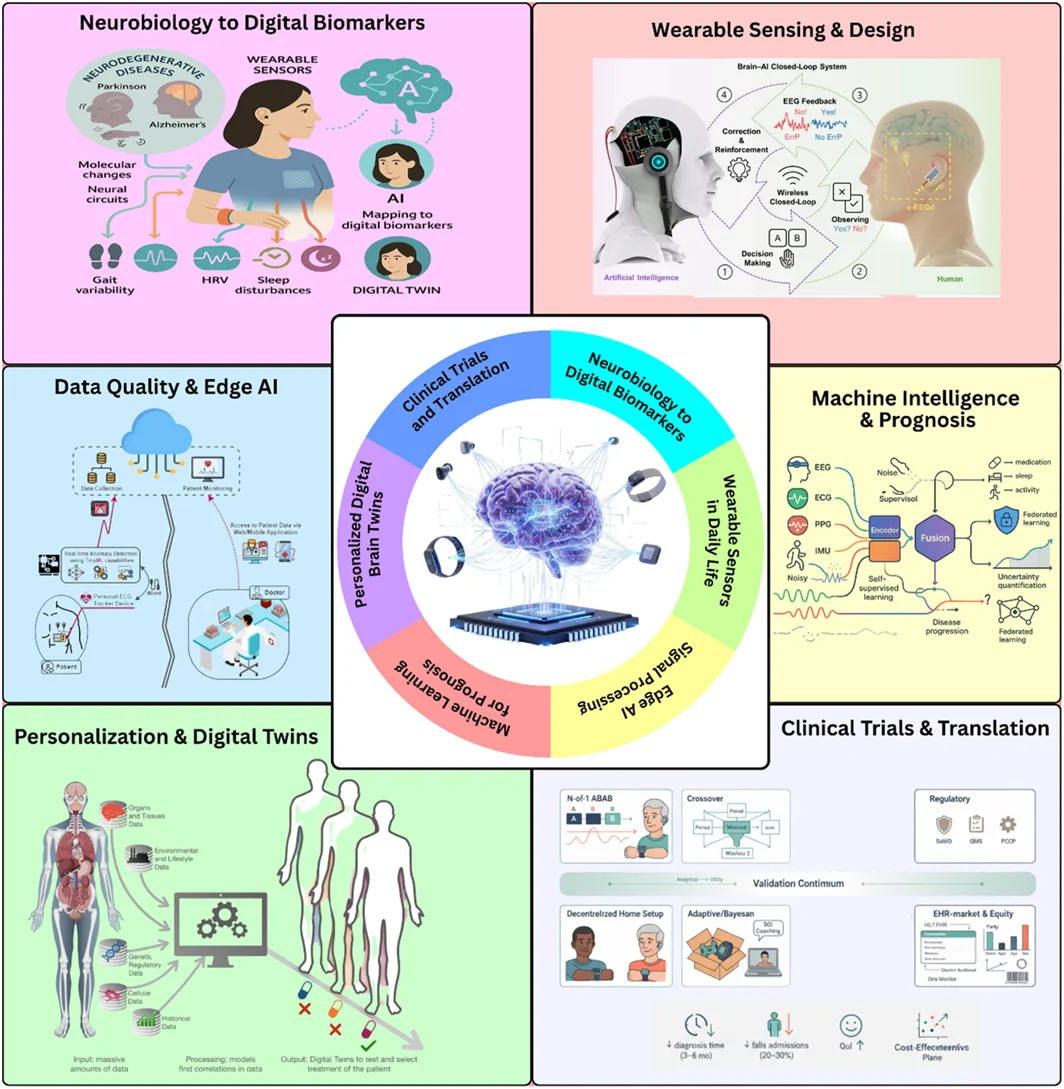

## 1 Introduction

Neurodegenerative diseases, including Alzheimer's disease and Parkinson's disease, are among the fastest‐growing health challenges worldwide. Despite their devastating effects, these disorders often develop silently over many years before clinical symptoms become apparent. During this preclinical period, pathological proteins accumulate and progressively damage brain networks involved in memory, movement, and behavior, creating a critical but largely missed window for early diagnosis and therapeutic intervention [1, 2].

Traditionally, neurodegenerative diseases are monitored using brain scans, spinal fluid tests, and in‐clinic assessments, which are expensive, invasive, time‐consuming, and require extensive clinical infrastructures. Additionally, these methods collect data only for a short period, limiting long‐term monitoring of brain health [3]. To address these challenges, neuroscientists have shown increased interest in wearable devices owing to their advantageous features, such as flexibility, compact size, and suitability for long‐term monitoring. These sensors are capable of continuously recording physiological and behavioral signals, including gait patterns, heart rate, sleep quality, and vocal characteristics, without disrupting the individual's daily activities [4]. However, despite substantial development in brain‐monitoring wearable devices, their practical utility is hindered by limited data processing and power for fast computations in long‐term assessment [5].

Advances in deep learning and edge computing now address both constraints. Integrated sensor–AI systems have been demonstrated for long‐term ambulatory collection [6, 7]. Multimodal models that incorporate gait, sleep, cardiac, and speech signals estimate disease risk more sensitively than models relying on a single stream [8]. However, a critical gap remains between digital monitoring and clinical therapeutics. New research frontiers are needed to establish mechanistic links between wearable‐derived biomarkers, brain dysfunction, and targeted medical interventions, enabling wearable AI systems to not only detect disease earlier but also guide personalized prevention and treatment strategies.

Several recent reviews on brain health monitoring have been published by different groups that have examined parts of this landscape, including Sun et al. [9] reviewed digital biomarkers for Parkinson's disease, emphasizing how wearable and digital measures can support earlier detection and more frequent monitoring, but largely remained within a single‐disorder framing. Unnik et al. [10] surveyed remote monitoring approaches for ALS, from telehealth to sensor‐based wearables, and highlighted adoption barriers and the need for harmonized standards, but did not focus on modern representation learning or multimodal fusion strategies. Li et al. [11] reviewed AI approaches for early dementia detection using digital biomarkers, but did not address sensor hardware constraints and real‐world data quality challenges. Finally, Zhang et al. [12] provided a systematic review of federated learning methods for healthcare, directly relevant to privacy‐preserving training on wearable data. However, a comprehensive framework integrating disease pathophysiology, wearable sensing, multimodal AI, clinical validation, and real‐world deployment remains lacking. To address this gap, we present a unified framework linking biological mechanisms to digital biomarkers and clinical decision‐making. We argue that clinically meaningful digital biomarkers must be biologically grounded, reliably measured, robustly analyzed, and rigorously validated. This review focuses on Alzheimer's disease, Parkinson's disease, and TDP‐43 proteinopathies, including ALS, with particular emphasis on pre‐symptomatic detection, where intervention is most effective. Given the substantial differences in prodromal duration across these disorders, we highlight the need for disease‐specific, continuous monitoring strategies throughout the disease trajectory.

The review is organized to progressively connect neurodegenerative biology, wearable sensing, AI analytics, clinical validation, and implementation. It first examines how disease pathology produces measurable alterations in sleep, gait, speech, autonomic function, and ocular dynamics, and then assesses wearable sensing technologies and signal‐processing strategies. Advanced AI approaches, including self‐supervised learning, multimodal fusion, and fairness‐aware modeling, are then discussed alongside clinical validation, regulatory considerations, and cost‐effective deployment. Finally, we introduce the Personalized Brain‐Health Operating System (pBH‐OS), a privacy‐preserving framework for scalable brain‐health monitoring. By integrating disease mechanisms, wearable technologies, and AI‐driven digital biomarkers, this review outlines a roadmap for biologically grounded and clinically scalable monitoring of neurodegenerative diseases.

## 2 From Neurobiology to Digital Biomarkers

The clinical value of a digital biomarker ultimately depends on its biological relevance. While wearable sensors can capture continuous physiological and behavioral data, these measurements are meaningful only when they reflect underlying disease mechanisms. Establishing clear links between neurodegenerative pathology, neural circuit dysfunction, and sensor‐detectable phenotypes is therefore essential for developing robust and generalizable biomarkers [13].

### 2.1 Pathophysiology‐to‐Phenotype Mapping

Three major neurodegenerative disease groups, including synucleinopathies, Alzheimer's disease (AD), and TDP‐43 spectrum disorders, each damage distinct neural circuits and produce characteristic patterns of functional decline. Table 1 summarizes the complete disease‐specific mapping from molecular pathology through circuit‐level dysfunction to wearable‐detectable digital phenotypes, including prodromal windows, sensor configurations, key confounders, and gold‐standard validation biomarkers.

**TABLE 1:** Quantitative wearable‐derived digital phenotypes across neurodegenerative disease stages.

Disease/stage	Digital measure	Quantitative finding	Sensor/reference	Citation
Prodromal + clinical PD	Seven‐day wrist accelerometry	AUPRC 0.14 ± 0.04 diagnosed; 0.07 ± 0.03 prodromal, ≤ 7 yr pre‐diagnosis	Wrist accelerometer/linked self‐report and health‐record diagnoses	[14]
Prodromal PD	Gait, pace domain	Deviates from non‐converters ≈ 4 yr pre‐diagnosis	Lower‐back accelerometer/clinical dx	[15]
PD detection	Nocturnal breathing	AUC 0.889; sens 80.22%; spec 78.62%	Breathing belt/clinical dx	[16]
PD, motor fluctuation	Real‐life gait, video‐referenced	Area under the receiver operating characteristic curve 0.82 for the most‐affected ankle; 0.84 using all sensors	Wrist, ankle, lower‐back, pocket sensors/video annotation	[17]
Isolated RBD	Cardiovascular autonomic function	Neurogenic OH → phenoconversion HR 5.05 (95% CI 1.39–18.38; *p* = 0.012)	Autonomic reflex testing/phenoconversion	[18]
Isolated RBD	HRV with deep breathing + MDS‐UPDRS III	87% abnormal at baseline; 6.6%/yr conversion; AUC 0.77 → 0.89 with disease duration	Neurologist‐confirmed phenoconversion	[19]
Incident all‐cause dementia	Daily step count and cadence	Optimal 9826 steps/day HR 0.49 (0.39–0.62); minimal 3826 steps/day HR 0.75 (0.67–0.83)	Wrist accelerometer/linked hospital, primary‐care, and death‐register records	[20]
MCI → dementia	Augmented‐reality iADL task	ROC‐AUC 0.91 at 3 yr; 0.94 in Aβ/tau‐positive aMCI	Smartphone/MMSE, MoCA, CDR	[21]
Prodromal–mild AD	9 acoustic + linguistic speech measures	Composite showed significant 18‐month change; effect size comparable to clinical endpoints	Speech from CDR interview/CDR	[22]
Amnestic MCI	Wrist‐sensor physiological + behavioral features	Neuropsychological scores predicted from sensor features; validated by leave‐one‐subject‐out and leave‐one‐interval‐out cross‐validation	Wrist‐worn wearable/neuropsychological battery	[23]

#### 2.1.1 Synucleinopathies (Parkinson's Disease and Dementia With Lewy Bodies)

Parkinson's disease and Lewy body dementia (LBD) begin when misfolded α‐synuclein proteins accumulate in the locus coeruleus and the pedunculopontine nucleus in the brainstem. These regions are responsible for regulating arousal, the autonomic nervous system, and posture. Damage to the pedunculopontine nucleus disrupts both postural and locomotor control, while degeneration of the adjacent sublaterodorsal (subcoeruleus) region disrupts the pontomedullary circuits that produce REM sleep atonia, thereby causing REM sleep behavior disorder (RBD). RBD involves a loss of the typical muscle relaxation during REM sleep, causing patients to physically act out their dreams. RBD may serve as the first sign during a prodromal phase that lasts 5–15 years before a clinical motor diagnosis of Parkinson's disease [24, 25]. Consumer‐grade Electroencephalography (EEG) and Photoplethysmography (PPG) sensors can detect decreases in heart rate variability (HRV) and motor events associated with RBD during sleep, acting as early, non‐invasive indicators of brainstem α‐synucleinopathy [26]. As pathology progresses to the substantia nigra, nigrostriatal dopaminergic denervation degrades basal ganglia motor circuit function. The resulting phenotype is characterized by increased stride‐time variability, increased arm‐swing asymmetry, prolonged turning duration, and the emergence of rest tremor [27]. The pattern of signal appearance, in which autonomic and sleep issues precede motor problems, mirrors the anatomical stages of α‐synuclein spread. This provides a biologically grounded framework for long‐term wearable monitoring [28].

#### 2.1.2 Alzheimer's Disease

Alzheimer's disease (AD) involves the accumulation of extracellular Aβ plaques and intracellular hyperphosphorylated tau neurofibrillary tangles. The initial impact of protein aggregates is on the entorhinal cortex and hippocampus, before they spread to the default mode network, which influences the cholinergic basal forebrain and other parts of the neocortex [29]. Amyloid‐induced hyperexcitability in the default mode network leads to heightened activity in the medial temporal lobe. This accelerates early tau accumulation, creating a feedback loop between amyloid and tau pathologies [30]. Preclinical Alzheimer's shows early signs detectable by wearable devices. These signs include reduced sleep spindle density, fragmented sleep‐wake cycles, and more speech pauses. These changes happen before memory complaints appear [31]. As the disease progresses and impacts frontoparietal oculomotor networks, the efficiency of smooth pursuit eye movements decreases, and saccadic latency increases. Concurrently, the decline in temporal and frontal language networks results in reduced verbal fluency [32]. These cognitive and oculomotor phenotypes differ from the motor‐dominant signature of synucleinopathies, highlighting the need for disease‐specific sensor configurations as detailed in Table 1.

#### 2.1.3 TDP‐43 Proteinopathies (ALS and Behavioral Variant Frontotemporal Dementia)

TDP‐43 (TAR DNA‐binding protein 43) is an important nuclear RNA‐binding protein that is necessary for the processing and transport of mRNA. TDP‐43 mislocalizes from its proper nuclear position to the cytoplasm in patients with ALS and behavioral variant frontotemporal dementia (bvFTD) [33]. This mislocalization leads to the formation of toxic aggregates via both gain‐of‐function (cytoplasmic toxicity) and loss‐of‐function (failure of nuclear RNA processing) mechanisms. The regional distribution of TDP‐43 pathology influences the clinical and digital phenotype [34].

In ALS, selective neuronal death in the corticospinal tract and spinal motor neurons leads to a measurable upper‐limb motor profile. This profile involves low peak hand acceleration, a high normalized jerk ratio (indicating movement irregularity), and altered agonist/antagonist muscle activation dynamics, as detected by surface electromyography (sEMG) [35]. In bvFTD, the salience network, especially the anterior insula and anterior cingulate cortex, is heavily affected. This reduces circadian amplitude, fragments daily rest‐activity rhythms, and causes behavioral disinhibition. The prodromal phase lasts 1–3 years for ALS and 3–8 years for bvFTD, requiring high‐frequency monitoring and sensitive detection algorithms [36]. A key complication is that TDP‐43‐related pathology frequently overlaps with amyloid and tau deposits in many AD patients, producing digital signatures that hinder accurate differential diagnosis [37]. Phenotypes such as low nocturnal HRV, disrupted sleep, and low activity are present in all three diseases. They are linked to depression, sleep apnea, and aging. Interpreting wearable data requires controlling variables such as medication timing, dosage, and comorbidities.

Additionally, Figure 1 integrates the three disease‐specific pathways discussed earlier into a single, clear visual framework. It illustrates how each proteinopathy begins at the molecular level, leads to circuit‐level disruptions, and results in digital phenotypes that can be detected via wearable devices, all mapped along the prodromal timeline. This diagram shows that the timing of signal appearance varies across diseases, which is important for selecting appropriate sensors and interpreting diagnostic results.

**FIGURE 1:**
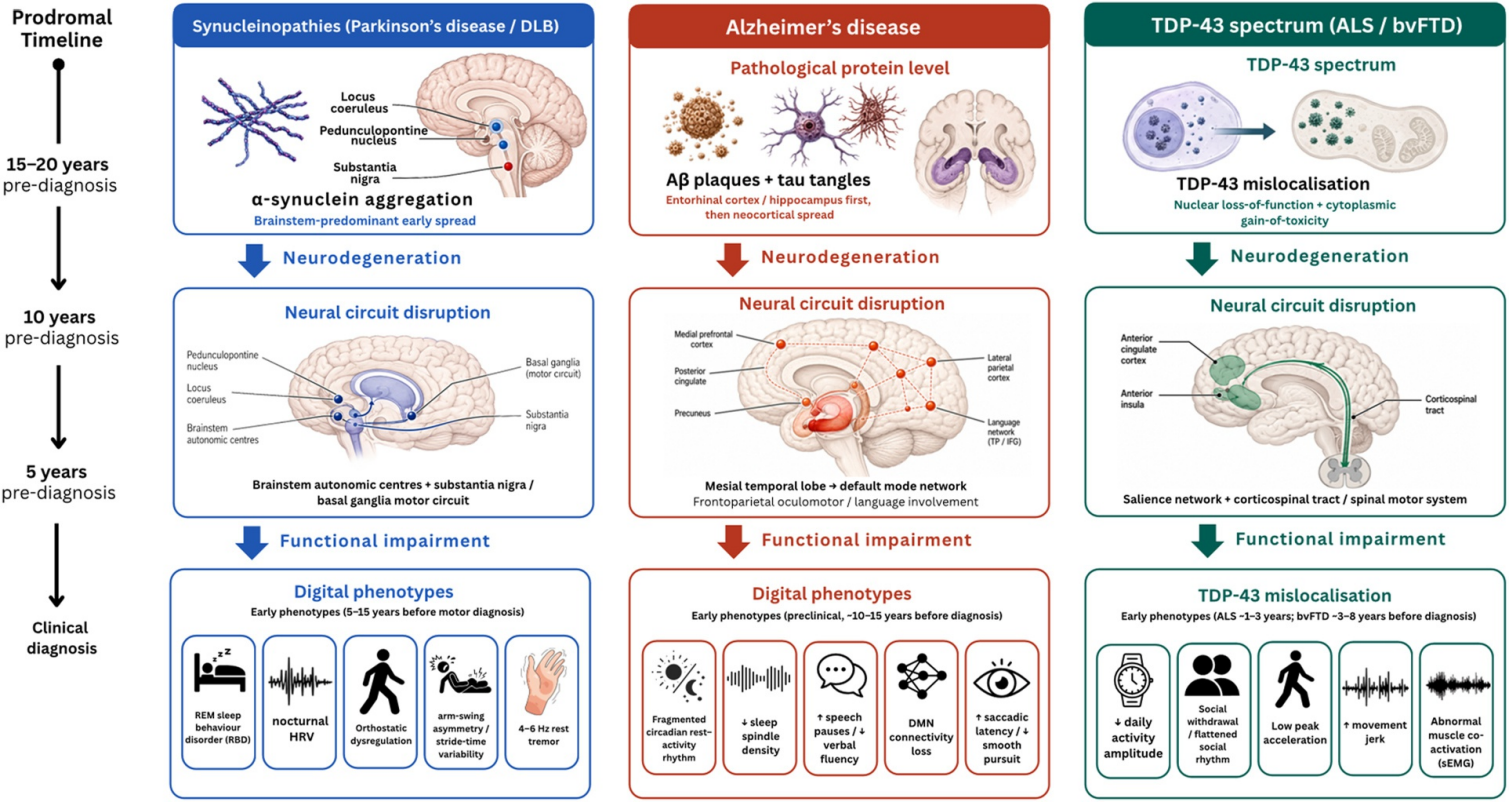
Pathophysiology‐to‐digital‐phenotype mapping across three major neurodegenerative disease groups. Each column represents one disease pathway: synucleinopathies (Parkinson's disease/dementia with Lewy bodies), Alzheimer's disease, and TDP‐43 spectrum disorders (ALS/behavioral variant frontotemporal dementia). The vertical axis represents the prodromal timeline from earliest detectable pathology (15–20 years pre‐diagnosis) to clinical diagnosis. For each disease, the figure traces the progression from pathological protein aggregation (top row) through neural circuit disruption (middle row) to wearable‐detectable digital phenotypes (bottom row). The distinct temporal ordering and phenotypic profiles across diseases highlight the necessity for disease‐specific, multimodal sensor configurations for early detection.

### 2.2 Prodromal and Wearable‐Tractable Signatures

The previous subsection identified the initial neural circuits impacted in various neurodegenerative diseases and the functional impairments seen in the prodromal stage. This section focuses on wearable sensing methods capable of detecting these impairments in real‐world, free‐living environments. Notably, sensor selection is grounded in biological relevance, with each sensor type linked to the specific biological system it monitors, as summarized in Table 2.

**TABLE 2:** Quantitative oculomotor, pupillary and speech‐language digital phenotypes across neurodegenerative pathways.

Disease/stage	Digital measure	Quantitative finding	Reference standard	Citation
Prodromal synucleinopathy (iRBD); de novo PD	Video‐oculography: Horizontal and vertical pro‐ and antisaccades	Antisaccade error rate raised versus controls in iRBD (*p* < 0.05) and de novo PD (*p* < 0.01); iRBD error rate correlated with MDS‐UPDRS III, Prague Stroop and Grooved Pegboard	PSG‐confirmed iRBD; MDS‐UPDRS, MoCA, neuropsychological battery	[38]
iRBD + early PD	Automated acoustic speech analysis: Composite dysarthria index (CDI), monopitch	CDI abnormal in iRBD (*p* = 0.002) and PD (*p* < 0.001); only monopitch separated iRBD from HC (*p* = 0.004); 12‐month progression iRBD *p* = 0.04, PD *p* = 0.03	Clinical diagnosis; blinded perceptual dysarthria score; MDS‐UPDRS	[39]
iRBD + early PD (free‐living)	Passive smartphone voice‐call acoustics plus active reading task	AUC 0.85 iRBD versus HC; imprecise vowel articulation in calls *p* = 0.03; monopitch in reading *p* = 0.05; 18 min (≈ 9 calls) sufficient for best sensitivity	PSG‐confirmed iRBD; laboratory high‐quality microphone recording	[40]
Clinical PD	Antisaccade error rate and latency, eye tracking	Error rate 0.34 (95% CI 0.30–0.39) versus HC 0.19 (0.16–0.22); *g* = 0.99 (0.76–1.21), *p* < 0.0001. Latency *g* = 0.84 (0.52–1.15), *p* < 0.0001; UPDRS‐III moderates latency (*R* ^2^ = 25.8%, *p* = 0.01)	Clinical PD diagnosis; UPDRS‐III, Hoehn and Yahr stage	[41]
Amnestic MCI (early AD risk)	Task‐evoked pupillary dilation during digit‐span recall	Amnestic single‐domain MCI showed greater dilation than non‐amnestic MCI and controls at lower cognitive loads despite equivalent recall; multi‐domain MCI failed to modulate dilation and performed worse	Jak–Bondi neuropsychological MCI classification	[42]
Preclinical AD risk (midlife, cognitively normal)	Task‐evoked pupillary dilation, digit span (locus coeruleus–noradrenergic proxy)	Heritability *h* ^2^ = 0.30–0.36; higher AD polygenic risk associated with greater dilation at 9‐digit load, Cohen's *d* = 0.36 (upper vs. lower AD‐PRS quartile); held after APOE adjustment	AD polygenic risk score; APOE genotype	[43]
MCI and dementia	Natural‐language processing of automated transcripts of digitally recorded neuropsychological exams	Held‐out AUC 92.6% (normal vs. dementia); 88.0% (normal or MCI vs. dementia); 74.4% (normal vs. MCI)	Framingham heart study consensus cognitive diagnosis	[44]
Presymptomatic TDP‐43 spectrum (ALS/FTD gene carriers)	Video‐oculography: Antisaccade and delayed‐saccade error rate, saccadic intrusions	Antisaccade error 21% (IQR 13–41) versus 15% (6–30) in controls, *p* = 0.0091; *C9orf72* versus controls *p* = 0.0242; error rate versus ECAS total in *C9orf72 r* = −0.74, *p* = 0.009; unchanged at follow‐up	Genetic testing; ECAS	[45]
ALS, early/middle to incomplete locked‐in state	50 Hz remote eye tracking: Prosaccade latency, antisaccade error, smooth‐pursuit gain	Antisaccade error group effect χ^2^(2) = 35.44, *p* < 0.001; pursuit gain χ^2^(2) = 39.20, *p* < 0.001; bulbar versus spinal onset made more horizontal antisaccade errors (OR 0.81, *p* = 0.034)	Revised El Escorial criteria; ALSFRS‐R	[46]
ALS and PLS, longitudinal	Eye tracking: Antisaccade, trail‐making, visual search	ALS impaired on executive and visual‐search tasks despite normal basic saccadic function; no reliable longitudinal change over approximately 20 months	Clinical ALS/PLS diagnosis; resting‐state fMRI connectivity (exploratory)	[47]
ALS‐FTD spectrum, bulbar involvement	Automated vowel acoustics from spontaneous picture description: Vowel space area (VSA), mean F2 slope	ALS + bulbar versus ALS‐nonbulbar |d| = 0.94 (VSA), 1.04 (F2 slope); versus bvFTD: 0.89 and 1.47; versus HC: 0.73 and 0.99. Correlated with bulbar score (VSA *R* = 0.33, *p* = 0.043; F2 slope *R* = 0.38, *p* = 0.011)	ALSFRS‐r bulbar plus Penn UMN bulbar subscore; MRI oral primary motor cortex thickness	[48]
bvFTD	Automated prosody: f0 range, speech‐segment and pause duration	Speech‐segment duration 1.33 ± 0.33 s versus 2.15 ± 0.64 s in HC (*p* < 0.005); pause duration 1.73 ± 0.86 s versus 0.94 ± 0.54 s (*p* = 0.0002); speech‐to‐pause ratio 1.02 ± 0.58 versus 2.84 ± 1.51 (*p* < 0.0001)	Consensus bvFTD criteria; matched healthy controls	[49]

Degeneration of brainstem autonomic nuclei, particularly the locus coeruleus and the dorsal motor nucleus of the vagus, disrupts heart rate control. This deterioration is detected using wrist photoplethysmography sensors and electrocardiogram monitors. These tools measure decreases in HRV metrics (RMSSD, pNN50) and the loss of orthostatic blood pressure recovery. They provide non‐invasive, early biomarkers for brainstem autonomic degeneration [50]. Degeneration of the basal ganglia, cerebellar, and cortical motor pathways leads to reduced gait automaticity and smoothness. Inertial measurement units (IMUs), including three‐axis accelerometers and gyroscopes, in wearable devices measure parameters such as stride‐time variability, arm‐swing asymmetry ratios, and the time to complete 180‐degree turns. They also quantify the normalized jerk from the lower lumbar spine or the distal limbs [51]. Dorsey et al. [52] proposed that wrist‐worn IMU‐derived tremor features can distinguish pathological rest tremor from physiological and postural tremor during unconstrained daily activities. The oculomotor system's brainstem, cerebellar circuits, and frontal eye field projections are vulnerable to neurodegeneration. This vulnerability leads to saccade delays, reduced pursuit gain, and square‐wave jerks, which are measured using infrared video‐oculography and electrooculography (EOG) electrodes [53].

Speech production involves the controlled movement of the respiratory, laryngeal, and articulatory muscles, all of which are vulnerable to neurodegeneration. Acoustic biomarkers such as pause‐to‐speech ratio, vowel space, pitch jitter, and amplitude shimmer can be measured with standard microphones. Early signs of Parkinson’s‐related dysarthria include hypophonia and limited prosody, while early Alzheimer's may show increased pauses and slowed lexical retrieval [22]. Autonomic, motor, oculomotor, speech, and pupillary responses reveal neural health over time. Symptom differences in synucleinopathies indicate varied α‐synuclein spread. Pupillary responses during tasks can signal early degeneration of the norepinephrine system, linked to the locus coeruleus [54].

Moreover, to provide a visual overview, Figure 2 presents the five sensing modalities discussed above within a unified framework for prodromal monitoring. The top row identifies the early vulnerable neural systems targeted by each proteinopathy. The middle row maps each system to its corresponding wearable sensing modality, the specific digital biomarkers extracted, and the underlying physiological rationale. The bottom row illustrates the longitudinal, free‐living monitoring paradigm required to capture within‐individual trajectories over weeks, emphasizing that phenotypic heterogeneity in prodromal neurodegeneration necessitates multimodal sensing rather than reliance on any single signal.

**FIGURE 2:**
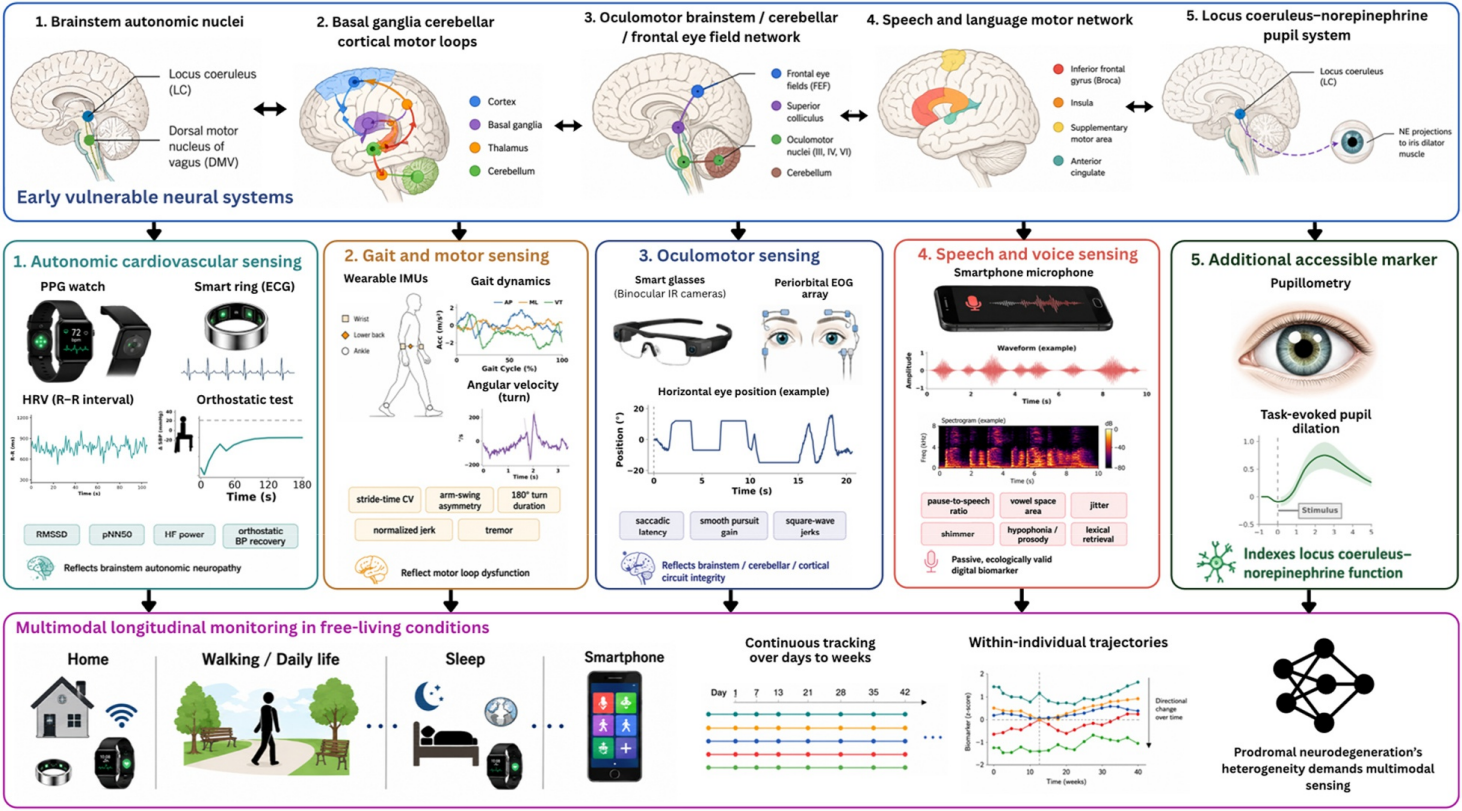
From vulnerable neural systems to multimodal wearable sensing in free‐living conditions. Top row: Five early vulnerable neural systems targeted across neurodegenerative proteinopathies: (1) brainstem autonomic nuclei, (2) basal ganglia–cerebellar–cortical motor loops, (3) oculomotor brainstem/cerebellar/frontal eye field networks, (4) speech and language motor networks, and (5) the locus coeruleus–norepinephrine pupil system. Middle row: The corresponding wearable sensing modalities for each neural system, including representative signal outputs and key extracted biomarkers. (1) PPG and ECG for autonomic HRV metrics; (2) IMUs for gait dynamics, including stride‐time variability, arm‐swing asymmetry, and tremor; (3) smart glasses and periorbital EOG for saccadic latency, smooth pursuit gain, and square‐wave jerks; (4) smartphone microphones for pause‐to‐speech ratio, vowel space area, jitter, and shimmer; (5) infrared pupillometry for task‐evoked pupil dilation indexing locus coeruleus–norepinephrine function. Bottom row: The multimodal longitudinal monitoring in free‐living conditions involves continuous data collection during home activities, walking, daily routines, and sleep over days or weeks. This enables tracking individual trajectories and detecting directional changes over time. The framework emphasizes that phenotypic heterogeneity in prodromal neurodegeneration necessitates multimodal sensing for effective early detection.

### 2.3 Ground‐Truth and Labeling Strategies

Detecting a disease‐relevant signal in wearable data is only meaningful if that signal can be paired with a reliable indication of the patient's disease state at the same time. Training AI models requires these paired examples, known as labeled data [55]. This subsection explains why obtaining reliable labels from continuous wearable recordings is difficult and describes the strategies that make it manageable.

The core problem is a density mismatch. Wearable sensors generate thousands of data points per day over months or years through continuous recording. Clinical assessments happen rarely, perhaps every few months, and produce a single score reflecting the patient's state during a brief clinic visit [56]. Established clinical rating tools, such as the Movement Disorder Society Unified Parkinson's Disease Rating Scale (MDS‐UPDRS) and the Montreal Cognitive Assessment (MoCA), capture performance at a single point in time and are sensitive to the patient's state on that specific day. Biological reference tests, such as dopamine imaging (DaTscan) and cerebrospinal fluid protein measurements, are more objective but invasive and expensive, and are performed only once or twice per study. The solution is not to choose one source of labels over another but to combine them strategically to build stronger supervisory signals for AI training [57, 58].

Three complementary approaches address this labeling challenge. Initially, temporal alignment limits the matching of wearable data with clinical labels around a clinical assessment. This approach helps minimize interference from daily symptom changes, resulting in more accurate sensor‐label pairings [17]. Second, semi‐supervised learning techniques extract probabilistic disease‐state labels from ancillary longitudinal data streams. These include clinical notes, health logs like falls and emergency admissions, medication records, and ecological momentary assessments (EMAs), brief questionnaires sent to patients' smartphones multiple times daily. Smets et al. [59] demonstrated that smartphone‐delivered EMAs provide high‐frequency labels for physiological data, thereby greatly increasing the number of labeled training examples. Third, active learning algorithms focus on data points with high uncertainty, meaning the model has low confidence in their classification or prediction, which reduces annotation effort and enhances the usefulness of each labeled example [60]. Consistency across devices, locations, and populations is essential. Sensor‐derived physiological measurements often exhibit systematic deviations due to variations in optical path geometry, analog‐to‐digital resolution, or skin‐sensor impedance. Domain‐adversarial neural networks mitigate this by learning device‐ and location‐invariant features, while invariant risk minimization further reduces center‐specific artifacts [61].

Standardized motor tasks at clinic visits, like a timed two‐minute walk or 30‐s finger‐tapping, serve as calibration anchors linking sensor recordings to clinical reference values [62]. Federated domain‐generalization frameworks allow cross‐institutional training without exchanging raw patient data, by aligning feature distributions from privately held datasets to a shared reference [63].

### 2.4 From Digital Phenotype to Validated Biomarker

The preceding subsections identified which neural circuits each proteinopathy damages first (2.1), and which wearable sensors detect the resulting functional changes (2.2). Section 2.3 addressed how continuous sensor recordings can be paired with verified clinical labels despite the fundamental mismatch between high‐density wearable output and sparse clinical assessment. Together, these subsections define what to measure, how to measure it, and how to anchor those measurements to disease state. A critical interpretive problem remains: many of the individual signals identified in Sections 2.1, 2.2 are not unique to a single disease. Reduced nocturnal heart rate variability, fragmented sleep architecture, and declining daily activity levels appear across all three proteinopathies. These features are equally characteristic of major depression, obstructive sleep apnea, chronic fatigue, and normal aging [64, 65]. On its own, no single wearable‐derived metric can differentiate prodromal Parkinson's disease from preclinical Alzheimer's or early‐stage ALS. This phenotypic ambiguity is not due to sensor limitations but is an inherent result of common downstream autonomic and sleep circuitry among different biological pathologies [66].

Resolution results from signals across different modalities. A decrease in HRV can indicate various conditions depending on accompanying factors. For instance, loss of REM muscle atonia on ear‐EEG, combined with gait asymmetry on wrist IMU, suggests brainstem synucleinopathy [67]. Conversely, HRV reduction alongside lower sleep spindle density and increased speech hesitations on a smartphone, without gait changes, points to early Alzheimer's. Minimal autonomic signs, such as rapid upper‐limb accelerations and movement irregularities on sEMG, are seen in TDP‐43 spectrum disorder. The key is recognizing the pattern and sequence of signals across modalities, rather than relying on a single metric [35, 68]. The temporal sequence of these conditions is driven by their pathogenesis. Autonomic and sleep dysfunctions, appearing 5–15 years before motor symptoms in synucleinopathies, are key early indicators. In Alzheimer's disease, sleep and language deficits precede ocular motor issues by several years. This necessitates a higher sampling frequency for significant detection [69]. Interpretation should account for variables beyond the disease that affect digital readings, including medication, biological rhythms, mental co‐morbidities, and environmental factors. These can cause systematic changes that mask the true pathology. Thus, monitoring systems need to record metadata such as medication timing, self‐reported mood, and ambient temperature to prevent confounders from being mistaken for neurodegeneration, reducing the risk of false alarms [70].

These biological and interpretive foundations now need to be translated into engineered systems. Whether the multimodal convergence patterns can be reliably captured in uncontrolled, real‐world conditions depends on various factors. These include sensor hardware specifications, anatomical placement, power limitations, and signal‐to‐noise ratio. Section 3 evaluates the wearable sensing platforms available to meet these requirements and the engineering trade‐offs that determine real‐world measurement fidelity.

## 3 Wearable Sensing Modalities and System Design

Wearable technologies are rapidly moving from devices that sense, process, and transmit physiological signals to practical clinical research. Modern wearable systems integrate diverse sensing modalities, including mechanical, electrical, optical, and biochemical sensors, to capture real‐time information about human health and performance. The synergy between advanced sensing strategies and intelligent system design is driving the development of more accurate, comfortable, and personalized wearable platforms for next‐generation healthcare [71, 72, 73].

This section evaluates existing signal measurement technologies applicable to mobile subjects under strict power and comfort constraints over extended periods. It outlines engineering trade‐offs that influence performance, presents quantitative targets for clinical viability, and consolidates them in Table 3. The table links each modality to its disease relevance, performance benchmarks, and validation standards. Given that synucleinopathies, Alzheimer's disease (AD), and TDP‐43 disorders exhibit distinct prodromal phenotypes (Section 2.1), sensor selection must be disease‐specific rather than technology‐driven. Each modality is associated with the disease pathway it is best suited to monitor, with Table 3 detailing the relevant pathway, validated performance, and reference standards.

**TABLE 3:** Quantitative acquisition and validation benchmarks for wearable sensing modalities in brain‐health monitoring.

Modality	Measured target/cohort	Wearable configuration	Acquisition specification	Reported quantitative validation	Citation
Scalp EEG	Resting EEG, VEP and P300; 16 controls + 16 memory‐clinic patients	Wireless headset; 19 dry electrodes	500 Hz; 24 bit; 0.04 μV LSB; < 2.0 μV p–p noise	Background classification *κ* = 1.00; P300 latency 388 versus 396 ms, dry/wet	[74]
Around‐ear EEG	Resting EEG and auditory P300; 10 adults	Two 10‐electrode cEEGrids; smartphone interface	500 Hz; 24 bit; DC–250 Hz; 7–8.3 h wear	P300 test–retest *r* ≥ 0.74; classification 70.34%–70.92%	[75]
fNIRS	Motor‐cortex HbO_2_/HbR; 8 adults	Backpack system; 8 emitters, 8 detectors, 20 channels	760/850 nm; 6.25 Hz; 25 ± 3 mm spacing; 2 h operation	HbR change 0.4 ± 0.2, 0.5 ± 0.2 and 0.3 ± 0.2 μmol L^−1^; *p* < 0.0025	[76]
Electrooculography	Ten saccade directions; 6 adults	Five dry foam electrodes in wireless goggles	256 Hz acquisition; 51.2 Hz classifier input	Mean classification accuracy 88.6%; best participant 93.0%	[77]
Mobile video‐oculography	Reading saccades and fixations; 11 adults	Binocular eye‐tracking glasses	60/120 Hz; 1000 Hz stationary reference	Saccades detected: 56.11 ± 12.44% versus 68.37 ± 13.97%; duration bias −10.81 versus −4.89 ms	[78]
Wrist PPG	Interbeat interval and heart rate; 30 adults	Empatica E4 wristband	PPG 64 Hz; reference ECG 500 Hz	Mean interbeat‐interval correlation *r* = 0.995–0.999	[79]
Lumbar IMU	Free‐living gait; 47 Parkinson's disease + 50 controls	Single 9 g triaxial sensor at L5	100 Hz; 16 bit; ± 8 g; 7 d	PD–control differences in 4/14 free‐living versus 2/14 laboratory gait metrics	[80]
Pressure insole	Gait ON/OFF medication; 21 Parkinson's disease patients	16 pressure sensors per insole plus 3D accelerometer/gyroscope	100 Hz; GAITRite reference 120 Hz	Cadence, speed and stride ICC = 0.93–0.97; phase‐time ICC = 0.48–0.57	[81]
Sweat cortisol	Sweat cortisol during acute stress	Microfluidic aptamer‐FET smartwatch	1 pM–1 μM range; 24‐bit ADC; 6 s sweep	Cortisol peaked 15 min after stress and returned to baseline by 90 min	[82]
Multimodal chest patch	Heart rate, HRV and respiration; 25 adults	Dry‐electrode ECG, four‐lead bioimpedance and 3‐axis accelerometer	ECG 256 Hz; Bio‐Z 32 Hz; accelerometer 64 Hz	Lin's CCC: Heart rate 0.98, HRV 0.85, respiration 0.56	[83]

### 3.1 Bioelectric and Hemodynamic Sensing: EEG, Ear‐EEG, and fNIRS

Scalp EEG, ear‐EEG, and functional near‐infrared spectroscopy (fNIRS) capture complementary aspects of brain activity. Understanding their respective strengths and limitations determines how they should be combined for neurodegenerative monitoring [84].

Scalp EEG measures voltage fluctuations generated by synchronized postsynaptic potentials. Clinically relevant signals span 0.5–45 Hz at amplitudes of 10–100 μV. Ambulatory systems sample at 250–500 Hz with at least 12‐bit analog‐to‐digital converter (ADC) resolution. Two feature families dominate in neurodegeneration research. Rhythmic features such as alpha power, theta‐to‐beta ratios, sleep spindles, and slow‐wave oscillations reflect the integrity of thalamocortical and cholinergic pathways affected early in AD [85, 86]. Aperiodic features, particularly the 1/f spectral slope, index the cortical excitation‐inhibition balance and shift measurably with cortical slowing. Both feature families require stable electrode contact for reliable extraction. Semi‐dry electrodes, skin preparation, strain‐relief cables, and active shielding reduce motion artifacts to acceptable levels [87, 88].

Ear‐EEG uses electrodes in the ear canal or concha, at the expense of spatial resolution but with the advantages of comfort and durability. Systems with 2–8 channels and impedance under 20 kΩ effectively record sleep slow oscillations, vigilance indices, and REM muscle tone, key signals for early synucleinopathy detection. The geometric constraints of the ear canal limit spatial coverage of the ear, making ear‐EEG well suited to sleep staging and aperiodic slope monitoring but not to spatial source localization. Cross‐ear bipolar referencing suppresses common‐mode noise more effectively than linked‐mastoid schemes when channel count is limited [89, 90].

fNIRS detects changes in cortical oxy‐ and deoxyhemoglobin concentration by measuring near‐infrared light absorption through the scalp [91]. Short‐separation channels (8–12 mm apart) are essential for measuring and regressing out superficial scalp blood flow, which would otherwise contaminate the cortical signal [92]. Hair, skin tone, and ambient light all attenuate signal quality; light‐tight optode housings and spring‐loaded contacts mitigate these effects [93]. When used together with wrist PPG or chest ECG, fNIRS helps distinguish cerebral hemodynamic responses from systemic cardiovascular variation. This is important for monitoring the nervous system, especially in aging populations. A nominal 3.7V, 500 mAh battery sustains continuous operation for approximately 50 hours at 10 Hz [94].

The current development of hardware is driving all three modalities toward clinical implementation. Shin et al. [95] reported a tattoo‐like EEG electrode, (Figure 3(I)), that fits the scalp, greatly minimizing motion artifacts due to improved electrode‐skin contact. Li et al. [96] proposed a multi‐module patch to overcome signal interference between EEG and fNIRS in multimodal sensing, as shown in Figure 3(II). The practical implementation of a miniaturized, high‐quality fNIRS system is demonstrated in the sensor design of Ma et al. [97], shown in Figure 3(III). Their design specifies the precise placement of devices and optical paths, with specific source‐detector separations and dual wavelengths. The basic principle of fNIRS measurement, including the importance of short separation channels for signal separation from the cortex, is clearly stated in the emitter‐detector arrangement diagram of Chen et al. [98], which is presented in Figure 3(IV). Finally, Figure 3(V) integrates low‐power data acquisition, processing, and wireless communication into a wearable device. It supports on‐device computation and day‐long operation for reliable, unobtrusive bioelectric monitoring.

**FIGURE 3:**
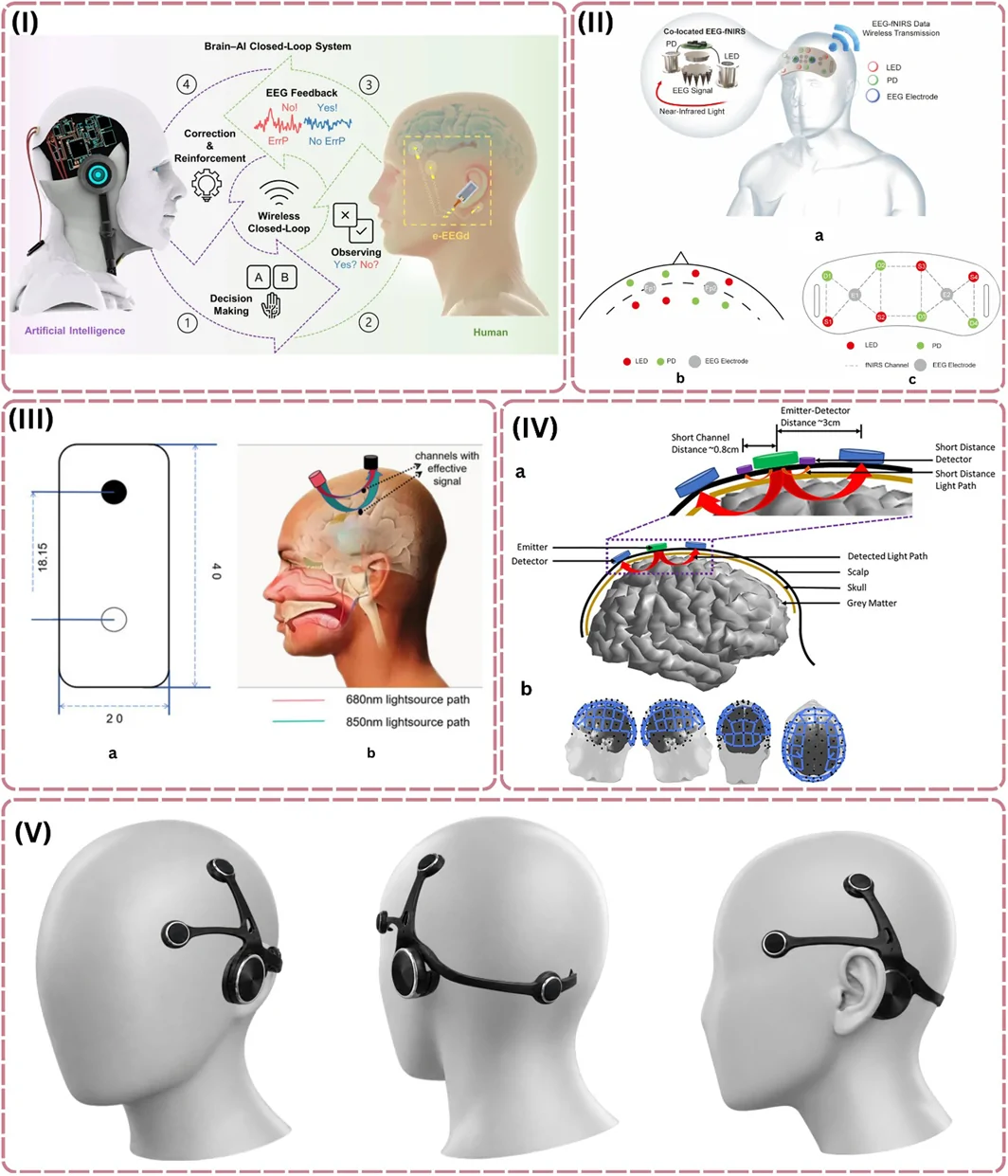
Advanced Sensor Architectures for Bioelectric and Hemodynamic Monitoring. (I) A brain‐AI closed‐loop system using tattoo‐like electronics for stable EEG acquisition. Reprinted under terms of the CC‐BY license [95]. Copyright 2022, The Authors, published by Springer Nature. (II) An integrated EEG‐fNIRS patch with an optimized layout to minimize crosstalk and enable synchronized measurement. Reprinted under terms of the CC‐BY license [96]. Copyright 2024, The Authors, published by MDPI. (III) A miniaturized fNIRS sensor design detailing emitter/detector placement and the intracranial optical path. Reprinted under terms of the CC‐BY license [97]. Copyright 2025, The Authors, published by MDPI. (IV) A schematic of fNIRS emitter‐detector arrangements and source‐detector separation for cortical and scalp signal measurement. Reprinted under terms of the CC‐BY license [98]. Copyright 2020, The Authors, published by Frontiers Media SA. (V) A conceptual design of an unobtrusive and ultra‐low‐power wearable EEG device.

### 3.2 Cardiopulmonary and Autonomic Sensing: PPG, ECG, HRV, EDA, and Respiration

Autonomic dysfunction often precedes the onset of motor symptoms in synucleinopathies, making it a valuable target for early monitoring. Wearable sensors such as photoplethysmography (PPG), electrocardiography (ECG), heart rate variability (HRV), electrodermal activity (EDA), and respiration sensors provide continuous insights into cardiovascular and autonomic nervous system function [42]. This subsection highlights these sensing modalities and the key requirements for reliable real‐world monitoring.

Photoplethysmography (PPG) monitors blood volume changes by measuring tissue light absorption. Green light (∼525 nm) is effective at the wrist and finger during daytime activity, while red and infrared light allow the estimation of oxygen saturation (SpO_2_) [99]. Electrocardiography (ECG) records cardiac electrical activity and provides more precise beat timing than PPG. Together, these signals support heart rate variability (HRV) analysis, the beat‐to‐beat variation in heart rate that reflects autonomic tone [100]. Standard HRV metrics include time‐domain measures (RMSSD, SDNN), frequency‐domain measures (LF/HF power ratio), and nonlinear indices (Poincaré plot geometry) [101]. Among the earliest digital indicators of synucleinopathy‐associated dysautonomia are reduced nocturnal RMSSD and attenuated 24‐h HRV circadian amplitude [102]. In AD, cholinergic degeneration disrupts cardiopulmonary coupling during sleep, producing a measurable alteration in the phase relationship between heart rate and respiration [103]. Baldelli et al. [18] reported that progressive deterioration of both sympathetic baroreflex sensitivity and parasympathetic cardiovagal responses occurs in patients with isolated REM sleep behavior disorder (iRBD). This finding establishes autonomic HRV measures as a primary prodromal biomarker for synucleinopathies. Respiratory rate can be estimated without a dedicated breathing belt by extracting PPG amplitude modulation and beat‐interval variation. Kalman‐filter fusion of rate estimates from multiple PPG channels yields robust results [104].

Electrodermal activity (EDA) provides another measure of autonomic arousal by quantifying variations in skin electrical conductance driven by sympathetic activity of the sweat glands. Distinguishing true sympathetic arousal from thermal artifacts requires separating the slow tonic component from fast phasic bursts and correcting for ambient skin temperature [105].

### 3.3 Neuromotor and Gait Sensing: IMUs, EMG, and EOG

Motor circuit integrity is impaired across all three disease pathways discussed in Section 2, resulting in quantifiable changes in walking, muscle activation, and eye movements. Sensors that measure these changes, and signal‐processing operations that transform the measurements into clinically interpretable outcomes, are discussed in this subsection.

Inertial measurement units (IMUs) integrate accelerometers and gyroscopes into low‐power, compact packages. Stride‐level gait analysis requires 50–100 Hz sampling; tremor detection requires 200 Hz [106]. IMUs mounted on the lower back or foot measure stride time, stride length, and turn time. They can estimate the stance phase of each step, and sensor drift is corrected via sensor fusion using zero‐velocity updates when the foot is stationary. The periodic nature of vertical acceleration and gyroscope signals enables robust step segmentation. Template‐matching classifiers use these signals to differentiate between walking, stair climbing, and riding in a vehicle [107, 108]. Shi et al. [109] reported a high level of accuracy (95%) in gait‐phase segmentation using foot‐mounted IMUs, confirming that gait‐phase segmentation can be used clinically for monitoring. In Parkinson's disease, increased stride variability and arm swing asymmetry detected by wrist IMUs, along with trunk gyroscope measurements, signal early impairment in synucleinopathies. Activity classifiers using integer‐arithmetic temporal convolutional networks efficiently distinguish walking, turning, and sitting, activating gait analysis only during relevant activities to conserve power [110, 111].

Surface electromyography (sEMG) records muscle electrical activity (20–450 Hz) using skin‐surface electrodes to detect rigidity, co‐contraction, and recruitment defects common in neurodegeneration. The sample rate of 1–2 kHz is sufficient to extract a reliable signal envelope. In conditions like ALS (TDP‐43 spectrum) and late‐stage synucleinopathies, sEMG is critical for tracking fasciculations and muscle tone abnormalities [112, 113]. Normalizing daily recordings with short‐term maximal voluntary contractions allows meaningful comparisons across sessions and individuals. On‐device feature extraction greatly reduces transmitted data compared to raw streaming [114].

Electrooculography (EOG) records saccades, smooth pursuit, and blink events by measuring the corneo‐retinal potential via spectacle‐mounted electrodes. Synucleinopathies are characterized by increased saccadic latency and slowed saccadic velocity; similarly, pursuit gain is demonstrably impaired in Alzheimer's disease [115]. These EOG‐identified abnormalities are precursors to neurodegeneration. Reliable ambulatory use needs brief calibration, blink differentiation, and drift compensation. Saccade measurement requires 200–500 Hz sampling. Head‐mounted IMUs record head motion alongside EOG, allowing gaze shifts to be separated from head movement [116]. Standardized short tasks (e.g., a 2‐min walk with turns, 30‐s finger tapping, and a reading passage) performed during clinic visits serve as calibration anchors. These anchors associate free‐living data with clinical references and establish test‐retest reliability across sites [117].

Surface electromyography (EMG) can be used to decode movements in high resolution, as reported by Kaifosh et al. [118] using a wearable platform shown in Figure 4(I). This 16‐channel, 2 kHz sEMG wristband measures rigidity, co‐contraction, and altered recruitment. The in‐ear EEG device (Figure 4(II)) studied by Moumane et al. [119] is an example of miniaturized biosensing for comfortable, long‐term wear. It is an example of miniaturized biosensing for comfortable, long‐term wear. The extensive diversity of wearable sensor form factors and their optimal placement across various body parts for comprehensive neuromotor assessment is well mapped by Zhao et al. [120], as shown in Figure 4(III). Zadeh [121] reviews wearable, handheld, and implantable biosensors for point‐of‐care diagnostics (Figure 4(IV)). biosensors for seamless, long‐term monitoring, addressing issues such as sensor placement and adhesion to improve data quality beyond traditional wearables. Wang et al. [122] (Figure 4(V)) propose “super‐resolution myography,” combining compact, high‐density EMG into muscle‐machine interfaces for healthcare and augmentation.

**FIGURE 4:**
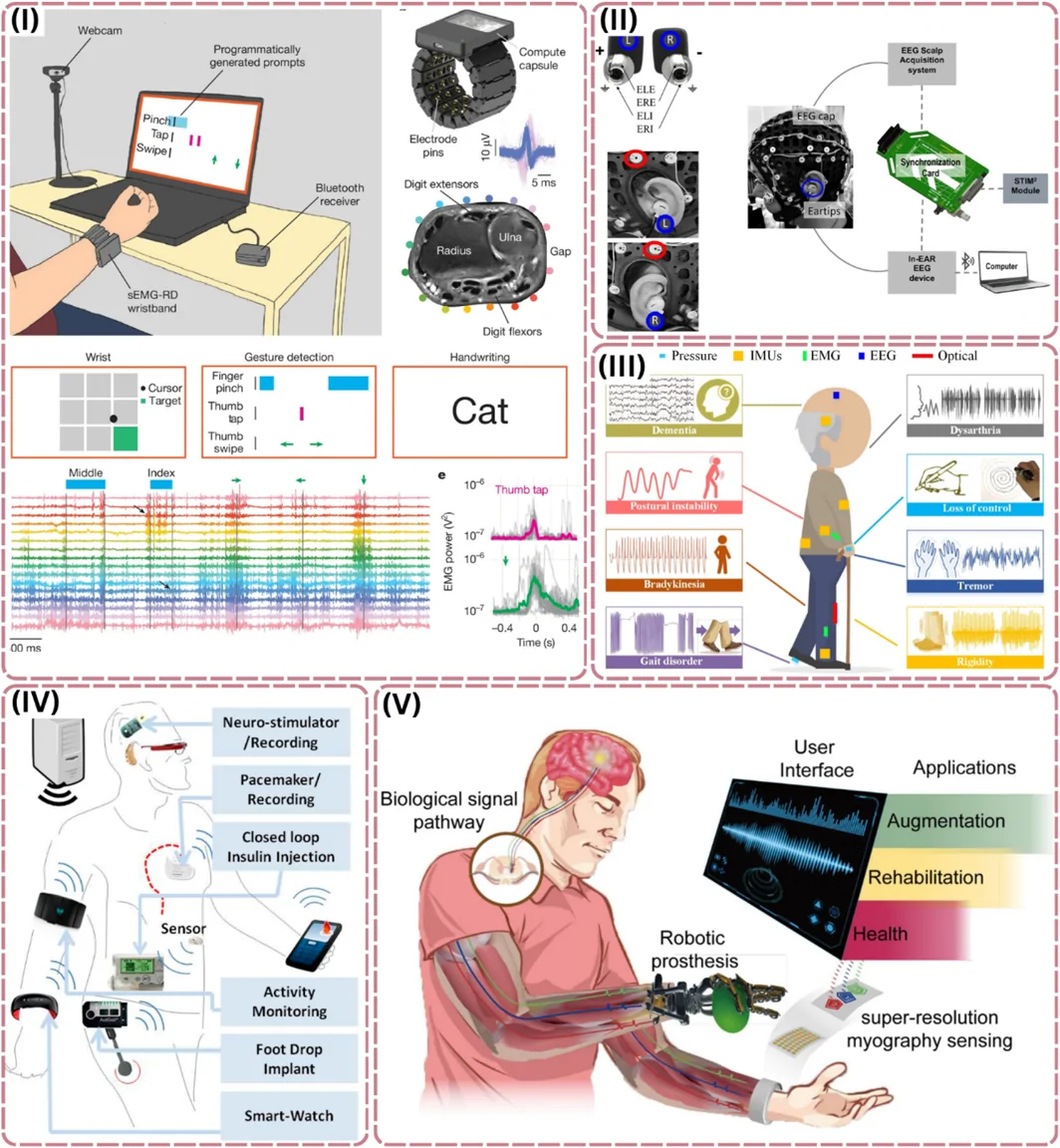
Wearable Platforms for Neuromotor and Electrophysiological Sensing. (I) A high‐throughput, dry‐electrode sEMG wristband for decoding hand and wrist movements. Reprinted with permission [118]. Copyright 2025, The Authors, published by Springer Nature. (II) An in‐ear EEG device with a cross‐ear bipolar electrode configuration for stable recording. Reprinted under terms of the CC‐BY license [119]. Copyright 2024, The Authors, published by Frontiers Media SA. (III) A taxonomy of wearable sensor placements on the human body for capturing comprehensive activity signals. Reprinted with permission [120]. Copyright 2023, The Authors, published by Sage Publishing. (IV) A classification of wearable, handheld, and implantable biosensor systems for point‐of‐care diagnostics. Reprinted under terms of the CC‐BY license [121]. Copyright 2015, The Authors, published by MDPI. (V) A conceptual schematic of wearable super‐resolution myography for muscle‐machine interface applications. Reprinted under terms of the CC‐BY license [122]. Copyright 2022, The Authors, published by Frontiers Media SA.

### 3.4 Smart Textiles, Electronic Skins, and Modular Multi‐Sensor Systems

Smart garments integrate conductive threads, printed electrodes and strain sensors directly into clothing. Textile ECG and EMG electrodes provide extensive contact on the skin and comfort in everyday life. Knitted or printed strain gauges measure respiratory excursion and joint angle, while pressure arrays record postural variation. The main engineering requirements are washability and stability in cycle‐to‐cycle impedance [123]. Lyons et al. [124] evaluated seven textile‐electrode materials through resistance‐degradation testing over 30 washing cycles. They demonstrated that nickel‐ and copper‐plated polyester electrodes could record surface EMG signals with quality comparable to that of conventional Ag/AgCl electrodes.

Electronic skins are thin, conformable adhesive patches that include multiple sensing elements such as EEG, EOG, or EMG electrodes, skin‐temperature sensors, and microfluidic channels for sweat biochemistry. They are designed to be lightweight and ensure close contact with the skin, reducing motion artifacts. Low‐power analog front‐ends, including a low‐noise amplifier, filter, and analog‐to‐digital converter (ADC), operate at microampere currents, allowing multi‐day use without bulky batteries [125, 126].

Modular body‐area networks use multiple sensor pods at the ear, wrist, ankle, and sternum, communicating wirelessly and providing redundant coverage. During exercise, if wrist PPG data becomes noisy, an ear sensor maintains cardiac timing. If a limb IMU saturates, a trunk sensor ensures stability estimates. Hardware timestamps and periodic sync beacons limit timing drift to 1 ppm, suitable for sleep and autonomic signals [127, 128]. Data schemas should encode raw signals, feature summaries, signal‐quality indices, device placement, and firmware versions using standard vocabularies. The clinical representation is detailed in Section 4.4. Co‐located skin temperature adjusts PPG amplitude for vasoconstriction and helps isolate vasomotor changes due to fever or ambient heat.

The field deployment environmental requirements include IP67 ingress protection, sweat‐wicking and antimicrobial fabrics, and adhesives that bond securely and release comfortably [129]. Beyond rigid form factors, smart textiles and electronic skins offer conformal contact and long‐term comfort. These technologies integrate conductive threads or ultrathin elastomeric sensors directly into clothing or onto the epidermis. While they resolve the mechanical mismatch between rigid sensors and soft tissue, challenges remain in maintaining stable electrical contact during vigorous movement and managing sweat accumulation. Modular multi‐sensor systems meet the need for concurrent multimodal data collection by distributing specialized sensing nodes across the body. However, they introduce complex synchronization and power‐management challenges [130, 131, 132].

In summary, each neurodegenerative digital biomarker identified in Section 2 corresponds to a specific sensing solution with defined technical parameters: ear‐EEG for sleep and autonomic markers; IMUs for gait; sEMG for motor rigidity; PPG/ECG for HRV; and EOG for gaze abnormalities. However, continuous real‐world monitoring faces two primary hurdles. First, no single platform captures all signal domains, necessitating complex, modular multi‐site networks. Second, raw ambulatory measurements are invariably contaminated by motion artifacts, variable skin contact, and environmental noise, issues that hardware alone cannot resolve.

## 4 Data Quality, Signal Processing, and Edge AI

The clinical value of wearable systems depends not only on signal acquisition but also on data quality. Real‐world recordings are frequently affected by motion artifacts, poor sensor contact, thermal drift, and device synchronization errors [133]. This section focuses on methods for artifact mitigation, edge AI‐enabled signal processing, multimodal data synchronization, missing‐data handling, reproducible analytics, and the management of real‐world data loss in wearable systems.

### 4.1 Artifact Detection and Signal Quality Management

Ambulatory recordings are inherently noisy. A pipeline that applies global filters without first assessing signal quality retains artifact‐contaminated and clean segments alike. The correct order of operations is to compute modality‐specific quality indices first, and to accept or reject each segment before any further processing [134]. Key EEG indices involve electrode impedance and mains interference. For reliable nocturnal HRV analysis using PPG, a high signal‐to‐noise ratio and a sufficient AC/DC ratio are necessary to detect prodromal synucleinopathy. ECG gating relies on baseline stability and confident R‐peak detection. IMU data quality is assessed through saturation flags and gravity‐vector consistency, helping to identify loose straps and intervals recorded in vehicles, which should be excluded from gait analysis. After gating, preprocessing must preserve both the amplitude and the phase of the physiological signal. High‐pass filters with cut‐offs at or below 0.5 Hz remove slow electrode drift without distorting the delta and theta oscillations used for sleep staging [135]. Adaptive notch cancellation suppresses mains interference with limited spectral leakage. Median filtering removes isolated spikes, and wavelet decomposition removes larger impulsive noise while preserving oscillatory structure. In multi‐channel EEG, independent component analysis separates ocular and muscular contamination from the cortical signal [136]. For motion artifact in PPG and ECG, learned denoisers such as one‐dimensional U‐Nets outperform fixed filters when trained on well‐labeled data. Their generalization to other devices and populations remains unproven and requires prospective testing before clinical use [137].

Artifact patterns change as deployment conditions vary. Active learning helps maintain rejection accuracy by guiding experts to review only the segments where the model is least certain, saving time on re‐annotation. Combining skin temperature, EDA, and movement variability provides reliable on‐body detection, while orientation analysis can identify incorrect placement, such as an inverted wrist device or a loose chest band [138, 139, 140]. These pipeline changes produce measurable gains. Impedance gating combined with wavelet denoising raises the proportion of usable ear‐EEG recording minutes per night [141].

### 4.2 On‐Device Inference and Energy‐Constrained Computing

Edge inference runs the model on the wearable itself. Raw biosignals are never transmitted. Only features or risk scores leave the device. This preserves privacy and permits real‐time alerting without network dependence. The constraint is power. Meeting the power constraint requires model compression. Eight‐bit integer quantization with per‐channel calibration and quantization‐aware training reduces model memory approximately fourfold and inference energy by two‐to fourfold, with negligible accuracy loss [142]. Hardware‐aware mixed‐precision quantization, which assigns different bit‐widths to layers based on sensitivity, yields further savings. Knowledge distillation trains a compact student model to reproduce a larger teacher's outputs, halving multiply‐accumulate operations while preserving calibration [143, 144].

Adaptive sampling and duty cycling significantly lower wearable sensor energy costs. In PPG acquisition, predictive sampling targeting key waveform regions cut power use by over 87%, with heart‐rate variability timing errors under 3 ms [145]. In inertial gait monitoring, adjusting sampling rate, window length, and sensor configuration can reduce data volume by up to 16 times while maintaining classification accuracy [146].

Hardware‐software co‐design provides additional improvements. Matching the analysis window length to the radio transmission period reduces wake‐ups. Co‐optimizing kernels for the processor's SIMD width with tiled on‐chip SRAM decreases memory‐access energy. Executing feature extraction within a trusted execution environment incurs overhead, with idle power in the hundreds of microwatts [147]. Yan et al. [148] reported on‐device arrhythmia and activity recognition with power consumption as low as 2.52 mW.

Moreover, Table 4 provides a detailed summary of these aspects, presenting an overview of published edge‐AI deployments for clinical wearable biosignal tasks, highlighting compression techniques, hardware choices, latency, efficiency metrics, performance, and their relevance to neurodegenerative monitoring.

**TABLE 4:** Benchmark of published edge‐AI deployments for clinical wearable biosignal tasks.

Study	Clinical task and signal modality	Precision and compression	Deployment platform	Inference latency	Reported efficiency
Busia et al., 2025 [149]	Five‐class arrhythmia classification, single‐lead ECG	INT8; 8‐bit quantization with 15‐epoch quantization‐aware fine‐tuning (QAT)	GAP9 parallel ultra‐low‐power RISC‐V MCU	4.28 ms at 240 MHz	0.09 mJ per inference
Akbari et al., 2025 [150]	Binary AF detection using 40‐beat RRI windows derived from ECG R‐peak annotations	—	STM32L475VGT6 ultra‐low‐power MCU with arm Cortex‐M4 processor	143 ms per 40‐RRI window, reported as 143 ± 0 ms	3532 ± 6 μJ per inference; results report 24.70 ± 0.04 mW and 13.72 ± 0.02 mA at 1.8 V
Ingolfsson et al., 2024a [151]	Seizure detection by EEG, PPG and accelerometer fusion	INT8 quantization; QuantLab/DORY	GAP9 PULP RISC‐V MCU	5.56 ms	0.11 mJ/inference; 19.25 mW mean; 21.43 GMAC/s/W
Ingolfsson et al., 2024b [152]	EEG artifact rejection followed by seizure detection	8/16/32‐Bit tree storage; MCCP pruning	GAP9 PULP RISC‐V MCU	0.3 ms/4‐s window	7.93 μJ/window; 26.1 mW; ∼300 h estimated
Busia et al., 2025 [153]	Seizure detection, scalp EEG	Binary spikes; INT8 PTQ weights	SYNtzulu on Lattice iCE40UP5K FPGA	0.5 µs/timestep	4.55 nJ per inference
Bian et al., 2024 [154]	Motor‐imagery brain‐computer interface with on‐device adaptation, EEG	FP32	GAP9 at 370 MHz and 0.8 V	14.9 ms per inference; 20 µs per on‐device update	0.76 mJ per inference; 0.83 μJ per update; 15.6 kB memory footprint
Kavoosi et al., 2023 [155]	Five‐stage sleep staging, single‐channel EEG	INT8; QAT fine‐tuning	Arduino Nano 33 BLE, arm Cortex‐M4	1.6 s/30‐s epoch	50 kB model; up to 280× smaller
Liu et al., 2023 [156]	Five‐stage sleep staging, 30‐s single‐channel EEG	—	Android NCNN; Snapdragon 865 CPU	2.8 ms per 30 s epoch	∼100 kB model; 48.95 M FLOPs/epoch
Hou et al., 2023 [157]	Freezing‐of‐gait detection with auditory alert; ear‐EEG, leg EMG and accelerometry	INT8 + pruning; > 2 MB to < 830 kB	nRF52840 around‐ear node; custom 180‐nm acquisition/UWB ICs	< 2.3 s/3‐s window	17 mA central node; 11.8 mA MCU; 0.74 mA/patch
Slemenšek et al., 2024 [158]	Freezing of gait detection and vibratory cueing; IMU and muscle activity	FP32 TensorFlow Lite	Portenta H7 STM32F747; 57‐g detector	261 ms	550 mW; 6 h
Al‐Adhaileh et al., 2025 [159]	Freezing‐of‐gait detection from multimodal wearable sensors	INT8 post‐training quantization with pruning; 6.5 MB reduced to 2.9 MB, a 54.2% reduction	Google Coral edge TPU and Raspberry Pi 4 (Cortex‐A72)	75 ms per window on Coral; 211 ms on Raspberry Pi 4; below 350 ms end to end	Model size reduced 54.2%; below 150 MB memory on Raspberry Pi 4

Abbreviations: AUC, area under the receiver operating characteristic curve; FP32, 32‐bit floating point; GMAC/s/W, giga multiply‐accumulate operations per second per watt; INT8, 8‐bit integer; MCU, microcontroller unit; MFLOPs, million floating‐point operations; PULP, parallel ultra‐low power.

### 4.3 Multimodal Alignment, Missingness, and Imputation

Combining multiple sensor streams requires accurate temporal synchronization and robust handling of missing data, both of which are common challenges in real‐world wearable deployments. Clock drift between devices can introduce timing errors that compromise cross‐modal analyses. To maintain synchronization errors below 50 ms, wearable systems typically employ hardware timestamping, periodic synchronization beacons, and post‐processing correction using a hub or smartphone reference clock [160]. Balasubramanian et al. [161] implemented this method, reducing the synchronization error from approximately 30 to 0.356 ms, with a 97.3% success rate.

Registering IMU cycles with ECG or PPG R‐peaks offers a secondary calibration and minimizes alignment errors. Short gaps can be filled with interpolation, while longer gaps are reconstructed using autoencoders that provide uncertainty estimates. These estimates inform risk scores, which should account for imputation confidence. Applying this method to longitudinal data reduced day‐to‐day gait variability without compromising statistical power [162, 163].

The fusion strategy should be selected based on which modality is most frequently dropped. Segment‐level fusion merges independently computed features for each modality, making it relatively resilient to modality loss. Sequence‐level fusion models interact across modalities over time, but are more vulnerable to missing data and need explicit masks for gaps. A practical solution involves calculating quality‐weighted features for each segment at the edge, then using a simple sequence model on the hub that processes missingness masks and quality scores together with the features [164, 165].

### 4.4 Standardized Pipelines, Reproducibility, and MLOps

A digital biomarker that cannot be reproduced by an independent group on a different dataset has no clinical value, regardless of its performance in the original study. Reproducibility requires that every processing step be explicitly specified, versioned and portable. Each pipeline should be specified by a declarative configuration file in YAML or JSON that defines the full transformation from raw sensor data to biomarker output [166]. All steps should be containerized, with dependency versions explicitly pinned and random seeds fixed. Raw data must be stored as immutable, read‐only objects verified through cryptographic checksums. Derived datasets should include manifests that document the software version, hyperparameters, and the time‐based train‐test split. This helps mitigate a common source of data leakage, where future data inadvertently influences model training. Dataset documentation should include cohort demographics, device models, signal coverage, and known limitations to enable external reviewers to assess generalizability [167, 168].

Deployed pipelines require ongoing monitoring to detect data drift, which can result from aging hardware, shifts in patient populations, or changes in environmental conditions. This process involves assessing the stability of feature distributions at the population level. If the drift exceeds a predefined threshold, the pipeline should be halted for review prior to delivering results to clinicians. New model versions should be initially deployed to a small canary cohort and then shadow‐tested against the current production model, with rollback procedures established in advance [169, 170].

Clinical integration involves linking the pipeline outputs to electronic health record systems. HL7 FHIR Observation resources carry LOINC codes together with biomarker values, device metadata and signal quality indices. Clinician‐facing dashboards should display data coverage, quality trends and model update history alongside the risk score, so that a clinician can judge whether a score rests on sufficiently high‐quality data before acting on it [171, 172]. Satagopam et al. [173] demonstrate this pattern in a translational medicine setting, integrating raw data management, analysis workflow, and clinical visualization into a single pipeline.

### 4.5 Real‐World Data Yield: Adherence, Device Dropout, and Missingness in Practice

The preceding subsections assume a data stream that is noisy but present. In practice, the dominant source of missing data is not artifact but non‐wear. Any pipeline specification is incomplete without a stated tolerance for the yield it will actually receive.

Adherence declines predictably over time and with increased device burden. Wrist‐worn and ring‐based devices demonstrate the highest compliance levels, attributable to minimal behavioral disruption, whereas chest straps and head‐mounted systems show the most rapid degradation in adherence [174, 175]. In neurodegenerative populations, reported compliance is highest in short, actively prompted protocols and decreases significantly during passive, multi‐month monitoring periods. Two longitudinal cohorts of Parkinson's disease patients maintained median wrist‐wear times of 21.1–21.9 hours per day over 2–3 years, provided that devices were comfortable, compliance was monitored, and proactive support was implemented [176]. Cognitive impairment exacerbates this issue. Patients with prodromal Alzheimer's disease may forget to charge or replace a device, and caregiver‐supported charging schedules significantly alter the observed yield. Motor impairment affects it differently; patients with advanced Parkinson's disease may be unable to fasten a strap unaided during an off period, resulting in systematic data loss correlated with symptom severity [177]. Missingness in neurodegenerative monitoring is rarely missing at random because the periods when a patient is least able to wear a device are often the periods of greatest clinical interest [178].

There are three key considerations for pipeline design. First, wear‐time metrics should be reported as primary outcomes instead of optional supplementary information. These include analyzable days per participant‐month, median daily wear hours, and the percentage of nights that meet sleep‐analysis quality standards [179, 180]. For example, in their Bellerophon‐based analysis, Tackney et al. [181] explicitly defined a compliant day as at least 10 hours of waking wear and a compliant month as at least 14 compliant days. Second, missing data should be modeled explicitly rather than automatically filled in. Since non‐wear periods are informative, analyzing only complete cases can bias results toward healthier parts of a participant's week. Naive imputation can worsen this bias. Third, it is important to distinguish between participant dropout and non‐wear episodes. Withdrawal is a censoring event to be modeled in survival analysis. Device removal lasting multiple days is a gap to be masked. Confusing these can artificially inflate perceived progression rates. Little et al. [182] distinguished treatment discontinuation from loss of outcome follow‐up and recommended continuing outcome collection after treatment withdrawal whenever possible.

Moreover, Figure 5 presents an integrated pipeline for converting heterogeneous wearable signals into clinically interpretable brain‐health indicators. It highlights the key requirements for reliable ambulatory monitoring, including signal‐quality control, artifact removal, on‐device inference, temporal synchronization, multimodal fusion, and continuous system evaluation.

**FIGURE 5:**
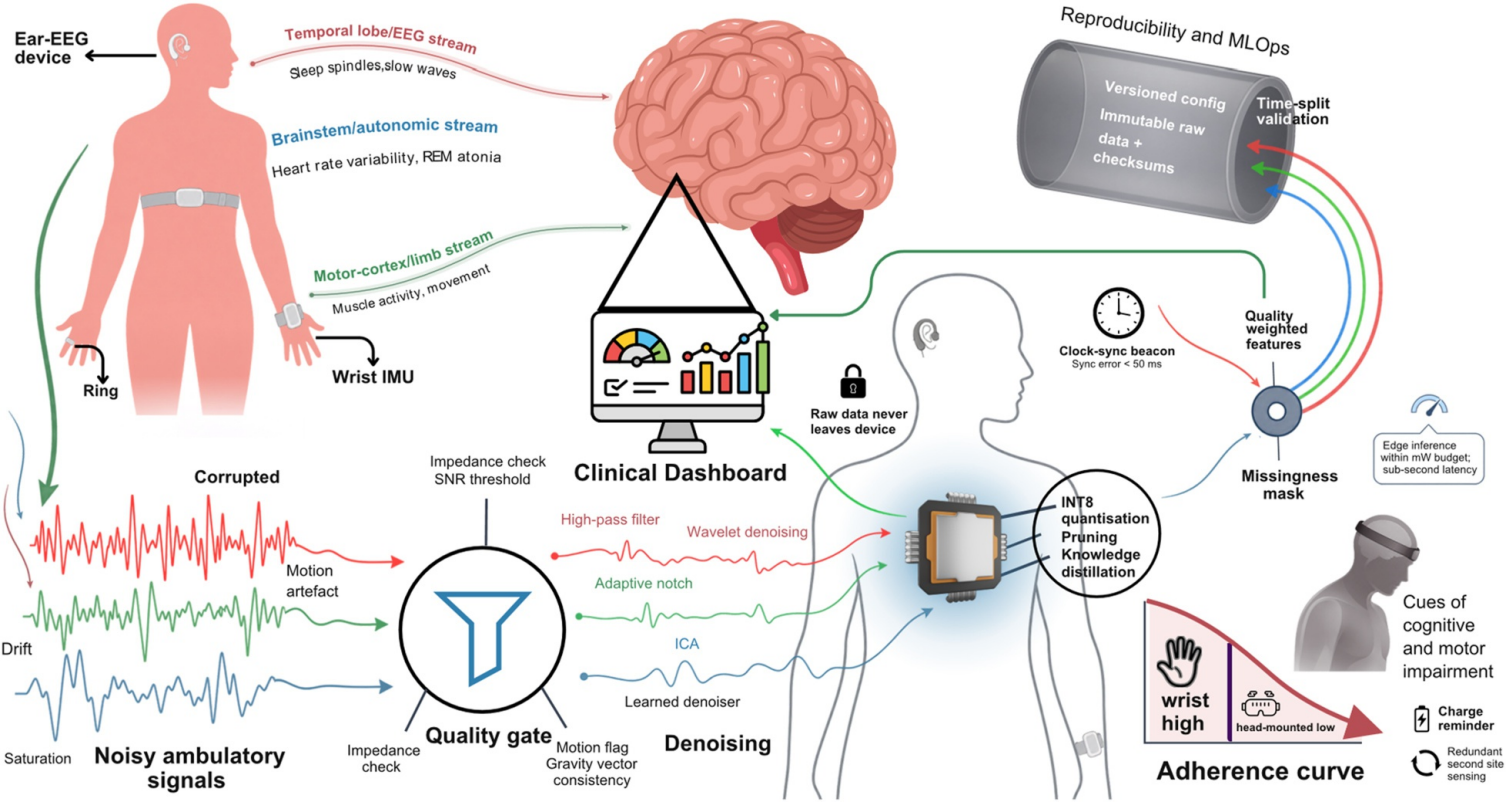
Data quality, signal processing, and edge AI pipeline for wearable brain health monitoring. Multimodal signals acquired from neural, autonomic, physiological, and movement sensors first undergo modality‐specific quality assessment, artifact detection, and denoising. Valid signal segments are processed locally using compressed edge‐AI models, allowing derived features and risk scores, rather than continuous raw data, to be transmitted. Clock synchronization and missingness‐aware fusion support reliable integration across devices, while adherence monitoring, uncertainty estimation, and version‐controlled MLOps maintain analytical robustness. The resulting longitudinal biomarkers can support clinical interpretation, individualized risk assessment, and early detection of neurodegenerative changes.

## 5 Artificial Intelligence for Early Detection, Prognosis, and Mechanistic Insight

Wearable devices generate large volumes of continuous, multimodal physiological data that require advanced computational methods to extract clinically meaningful insights. The key challenge is to identify robust physiological patterns that enable early disease detection, prognosis, and treatment monitoring [183]. This section discusses self‐supervised representation learning, multimodal data fusion, causal modeling for improved interpretability, and federated and continual learning frameworks for privacy‐preserving deployment. It also highlights the current limitations and future challenges of these approaches.

The applicability of the four strategies varies across different disease groups. Self‐supervised pretraining demonstrates particular utility in Alzheimer's disease, where extensive unlabeled sleep‐EEG and speech datasets are available, but validated preclinical labels remain limited [184]. Multimodal fusion is especially beneficial for synucleinopathies, as diagnostic information is embedded in the combined patterns of autonomic, sleep, and motor alterations rather than within any single modality [185]. Causal modeling proves most advantageous for Parkinson's disease, given that dopaminergic medication acts as a significant and dynamic confounder affecting all motor and autonomic measures [186]. Federated learning is most pertinent to TDP‐43 spectrum disorders, where individual research sites face challenges in assembling sufficiently large cohorts for model training, and the narrow prodromal window constrains local data accumulation [187].

### 5.1 Self‐Supervised and Foundation Models for Biosignal Representation

Most wearable datasets are unlabeled, and the clinical assessments needed to label them are infrequent, costly and inconsistently administered. Self‐supervised learning (SSL) addresses this by training on the internal structure of the data rather than on external labels [188].

SSL approaches are widely used for biosignals: contrastive learning, which brings representations of similar signal segments closer while separating unrelated ones, and masked prediction, which reconstructs missing signal portions from the surrounding context. Both methods perform well with EEG, IMU, and PPG data when training augmentations preserve physiological relevance. Techniques such as time warping and amplitude jittering are suitable, whereas excessive spectral filtering can remove clinically important signal features [189, 190]. Pan et al. [191] applied physics‐preserving augmentations to unlabeled EEG and EOG, producing representations that transferred effectively to sleep staging. These modalities is most relevant to prodromal synucleinopathy and Alzheimer's disease. Foundation models enhance SSL by pretraining a single large encoder across diverse modalities, devices, and populations, and then fine‐tuning it for specific tasks with limited labeled data. The encoder processes biosignals into short segments and develops shared representations through prediction and denoising objectives [192]. Cross‐modal pretraining, such as predicting ECG features from PPG, aligns representations across sensor types and reduces performance gaps across devices, with AUROC differences decreasing after calibration [193].

### 5.2 Multimodal Fusion Architectures

Neurodegenerative diseases manifest through diverse physiological changes, including sleep disturbances, autonomic dysfunction, gait impairments, speech abnormalities, and oculomotor deficits, which evolve at different rates and reflect distinct neural pathologies. Consequently, effective multimodal fusion frameworks must integrate heterogeneous sensor streams, preserve temporal relationships, and remain robust to missing or unavailable modalities during real‐world monitoring [194, 195].

Three fusion strategies form a progression in complexity and robustness. Early fusion concatenates raw per‐modality features into a single model input; it is simple to implement but fragile, as the failure of any one sensor corrupts the entire input representation. Late fusion encodes each modality separately and combines the encoded outputs in a small integration network, so each stream contributes independently, and modality loss is tolerated. Hybrid fusion adds cross‐attention between modality‐specific encoders, allowing one stream to condition the interpretation of another. For example, it lets the sleep encoder modulate how the gait stream is read, while retaining independent processing paths [196]. Mostafaei et al. [197] showed that a transformer with cross‐modal attention outperformed both early and late fusion baselines for sleep staging on multimodal physiological data.

Fusion must also reconcile timescales. Short windows of 1–10 seconds are required to detect saccades, tremor bursts and heart‐rate transients. Contextual patterns such as circadian rhythm, sleep architecture and medication effect operate over minutes to hours. Temporal transformers with a hierarchical windowing operate across both timescales concurrently and are therefore well‐suited to neurodegenerative monitoring [198, 199]. Body‐area sensor network data can be represented as graphs and processed with graph neural networks (GNNs). The sensors form a graph, with each sensor being a node and each functional or physical relationship between sensors being an edge. GNNs also allow the model to discover what sensor pairs give complementary information on a particular task [200].

Cross‐device performance loss remains a major obstacle. Measurements from two wrist devices differ systematically even when they record the same signal at the same site. Bent et al. [201] evaluated six wrist‐worn photoplethysmography devices against electrocardiography in 53 participants and found significant device‐dependent measurement errors. Interpretability is also required during clinical deployment. Attention rollout methods examine which time windows and modalities had the strongest effect on a prediction. Concept activation vectors ensure that the model's internal representations are consistent with known physiological activity. For example, they verify that decreases in slow‐wave sleep and increases in stride variability contribute to Parkinson's risk scores in the predicted direction [202]. Moreover, Table 5 evaluates multimodal fusion studies based on accuracy, computational cost, and interpretability.

**TABLE 5:** Performance, compute and interpretability trade‐offs across multimodal wearable‐fusion studies.

Modalities used	Fusion/temporal handling	Primary reported performance	Efficiency/deployment/interpretability metrics reported	References
PPG + 3‐axis accelerometer	Early fusion: Time‐frequency spectra, 2D CNN	MAE 9.99 ± 5.9 bpm (PPG‐DaLiA) and 8.2 ± 3.6 bpm (WESAD)	26 K parameters, 190 K operations/s	[203]
PPG‐IHR + accelerometer‐ZCM	Hybrid/late: CNN + BiGRU per stream, then combine	Macro recall: 0.866 (2‐class), 0.832 (3‐class), 0.772 (4‐class), 0.671 (5‐class)	503 K params (IHR), 133 K (ZCM)	[204]
IHR (from wrist PPG) + activity counts (accelerometer)	End‐to‐end NN (conv + GRU)	4‐class median κ = 0.638, accuracy 77.8%	Runtime 0.206 versus 10.9 min/recording; 89.5M MAC ops/h	[205]
Wrist accelerometer + wrist PPG‐derived HRV	Feature‐level fusion: 132 HRV features + activity counts, dense + 3 biLSTM layers	Accuracy 76.4%, κ = 0.62 (epoch‐by‐epoch)	Signal acquisition noted: Accelerometer at 128 Hz, PPG at 32 Hz; feature set size: 132 HRV features.	[206]
pO_2_, HR, ACC, snoring	Feature‐level fusion into an ANN	Sleep detection: Sensitivity 91.5%, specificity 71.0%, accuracy 86.1%	ANN structure: 97 input features, 128‐neuron hidden layer	[207]
Temperature + EDA + ACC + PPG (BVP)	CNN on raw time‐series; modality fusion	Best overall fused performance: AUC‐ROC = 0.752	Cohort scale: 94 patients, 11,066 h sensor data	[208]
MRI brain parameters + wearable accelerometry + PRS + lifestyle	Feature‐level fusion into gradient‐boosted trees (XGBoost)	Best multimodal model AUC = 0.819; model without MRI AUC = 0.688	Feature importance: 18/20 top features MRI‐derived; 2/20 accelerometry‐derived	[209]
HRV, ACC, EDA + activity context	Multitask deep learning + SHAP	On SWELL‐KW burnout‐related tasks: 89% recall, 86% precision, *R* ^2^ ≈ 0.88	SHAP values reported (e.g., HRV SHAP 0.45)	[210]
Single‐lead ECG + IPG + motion	Multimodal deep learning + feature‐based	2‐class: Accuracy 83.6%, AUROC 86.0%; RCNN best F1 0.618 (3‐class) and 0.552 (4‐class)	Top 15 features retained 99% of full‐set performance and reduced training time by 73%	[211]
Wearable ECG + PPG	Feature fusion, mutual‐info, selection, random Forest	After calibration: MAE 5.21 mmHg (SBP) and 4.15 mmHg (DBP)	11 (SBP)/15 (DBP) features; device 42 × 29 × 13 mm	[212]

### 5.3 Causal Discovery and Counterfactual Risk Prediction

A correlation between a wearable biomarker and a clinical outcome does not necessarily indicate that the biomarker reflects the underlying disease process. Wearable signals are influenced by numerous confounding factors, including medication use, lifestyle behaviors, environmental conditions, comorbidities, and physiological variability. As a result, models trained solely on correlational relationships may inadvertently learn these confounders rather than disease‐specific patterns, leading to poor robustness and limited generalizability across populations and clinical settings [213, 214, 215]. Directed acyclic graphs provide a principled representation of assumed causal relationships between variables [216]. In a causal graph for Parkinson's monitoring, dopaminergic degeneration and dopaminergic dosing both influence nocturnal HRV. However, they act through different mechanisms; the graph makes this distinction explicit and estimable [217]. Chen et al. [218] showed that a two‐stage pipeline, selecting causally relevant features, then estimating their effect on progression, outperformed correlation‐based models under cross‐site validation.

A well‐specified causal model enables estimation of a patient's gait variability trajectory with respect to medication timing. Longitudinal data may include natural experiments such as dose‐timing changes, clinic closures, seasonal temperature shifts, and daylight‐saving transitions, which serve as exogenous shocks that enable causal inference without randomization [219]. Longitudinal data may include natural or quasi‐experimental variations, such as external service disruptions or policy shifts. Establishing causality requires a credible random assignment and control of confounding factors [220]. Li et al. [221] describe G‐Net, a recurrent neural network that uses a formal causal inference method, g‐computation, to predict the effects of dynamic treatment regimens in longitudinal studies. Physiology‐informed priors enhance causal models by integrating known relationships, such as the link between REM fragmentation and nocturnal HRV attenuation in synucleinopathies [222]. This approach encourages biologically realistic solutions and reduces false associations. Using differential equations to model slow pharmacokinetic processes, such as medication absorption, enhances the stability of longitudinal estimates. This is achieved by smoothing out short‐term fluctuations [223].

### 5.4 Continual, Federated, and Privacy‐Preserving Learning

A model trained on data from a single institution at a single point in time degrades as devices are updated, patient populations change, and new clinical sites are added. Clinical AI models must therefore adapt continuously without forgetting previously learned knowledge, and must do so without centralizing sensitive patient data. Continual learning mitigates catastrophic forgetting, which occurs when a neural network overwrites previous representations during training on new data [224]. Elastic weight consolidation (EWC) identifies parameters crucial to prior tasks and restricts their updates to preserve learned knowledge. Experience replay involves maintaining a condensed summary of past data and revisiting it during subsequent training sessions. Collectively, these approaches sustain AUROC within 1–2 points of baseline performance as new devices and populations are introduced over extended deployment periods [225]. Kim et al. [226] validated a continual learning framework for multi‐center ECG analysis that adapted to new sites without a central server and without forgetting previously learned arrhythmia patterns.

Federated learning enables multiple institutions to collaboratively train a shared model without transferring raw patient data. Each site trains locally and only sends encrypted weight updates to an aggregator, ensuring that raw signals, features, and identifiers are not shared [227]. FedProx manages heterogeneity in data quality and computational resources across sites by adding a proximal regularization term that keeps local models close to the global model [228]. FedBN keeps batch normalization parameters local to each site, preventing site‐specific distribution differences from corrupting the global model [229]. In practice, federated training across 5–10 hospitals achieves an AUROC that is 1–2 percentage points lower than that of centrally trained models while meeting data governance requirements [230].

Equity monitoring must be embedded in federated training, not added as an afterthought. Group‐robust loss functions penalize disproportionately large errors on underrepresented subgroups. For example, patients with darker skin tones, for whom PPG‐derived HRV is less accurate, or older patients with atypical gait patterns. Adaptive sample reweighting gives greater influence to rare subgroups during aggregation. Subgroup‐level calibration metrics with confidence intervals should replace aggregate AUROC as the primary reporting standard [231].

### 5.5 Current Limitations and Research Frontiers

This section examined four complementary strategies for extracting disease‐relevant information from wearable biosignals: self‐supervised representation learning, multimodal fusion, causal modeling and federated continual training. Together they address labeling scarcity, sensor heterogeneity, confounding and privacy constraints.

Several limitations continue to hinder clinical deployment. First, causal modeling relies on assumptions about unmeasured confounders, which may introduce bias and reduce the reliability of predictions. Second, SSL requires large, diverse datasets, which limits its effectiveness in small cohorts and rare neurodegenerative diseases. Third, federated learning often suffers from reduced performance under strict privacy constraints, particularly in small or heterogeneous populations, and the optimal balance between privacy and utility remains unresolved [232, 233].

The most effective approaches include multimodal foundation models trained jointly on biosignals and electronic health records, which facilitate transferability to new diseases and populations [234]. Additionally, online causal discovery methods can update causal graphs from streaming data without relying on batch assumptions. Federated meta‐learning also plays a key role by adapting to new sites with minimal local data [235].

The whole wearable‐to‐clinical‐insights conversion process is shown in Figure 6. Physiological data, continuously collected by sensors on different parts of the body, such as EEG, ECG, PPG, IMU, EOG, and smart textiles, are fed into a artificial intelligence system through the following five steps. The pipeline begins with the continuous collection of unlabeled multimodal data. Self‐supervised learning and cross‐modal attention integrate these streams, followed by causal modeling to separate disease progression from confounding factors. Federated learning enables privacy‐preserving training across multiple sites, ultimately generating personalized risk scores and interpretable alerts. The strategy involves transforming noisy, heterogeneous data from wearable devices to enable equitable, comprehensible tools for the early identification of neurodegenerative diseases.

**FIGURE 6:**
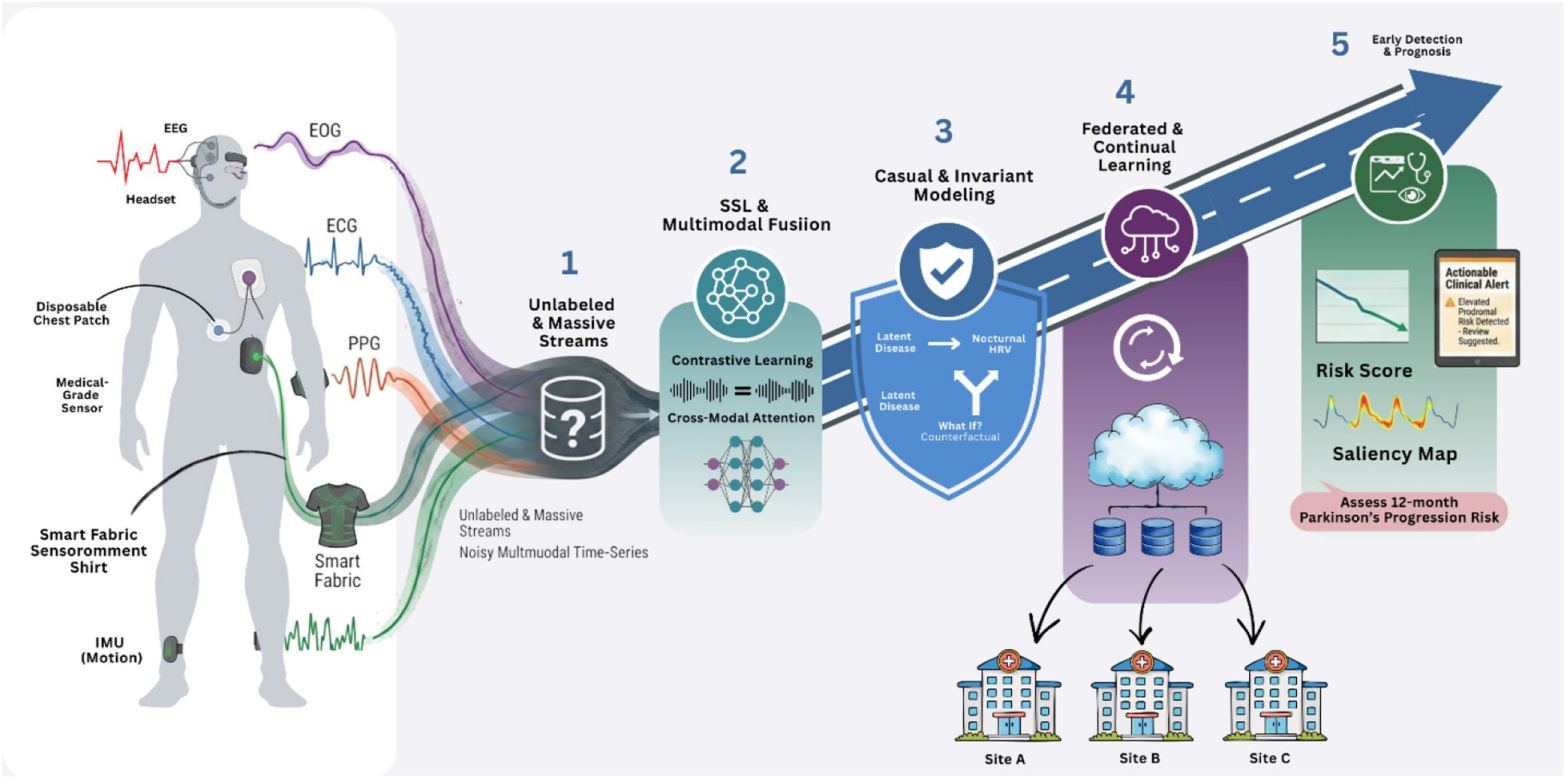
Artificial Intelligence Pipeline for Wearable Biomarker Discovery. Left: Multimodal wearable sensors (EEG, ECG, PPG, IMU, EOG, smart fabrics) capture continuous biosignals. Center: A five‐stage pipeline transforms raw streams into clinical insights through self‐supervised learning, multimodal fusion, causal modeling, federated training across sites, and early detection systems. Right: There are outputs of risk scores, actionable alerts, and interpretable saliency maps to evaluate prodromal Parkinson's disease.

## 6 Clinical Validation, Real‐World Trials, and Regulatory Translation

The greatest challenge in wearable healthcare is not building accurate algorithms, but translating them into tools that improve patient outcomes. Although AI models can extract disease‐relevant information from wearable data, clinical adoption requires far more than technical performance. Candidate digital biomarkers must demonstrate reproducibility, clinical utility, and regulatory compliance before they can support medical decision‐making [65]. This section explores clinical validation strategies, regulatory considerations, healthcare workflow integration, and implementation challenges, including patient adherence, dropout, missing data, and clinical accountability.

### 6.1 Trial Design for Wearable‐AI in Neurodegenerative Diseases

Wearable‐AI trials differ from conventional pharmaceutical trials in two respects. The outcome measures are continuous digital signals instead of discrete clinical assessments, and the diseases progress slowly and variably, so change is detected over years rather than weeks [178]. Statistical power calculations must be set per disease because prodromal duration and progression rate differ by an order of magnitude across the three groups. Synucleinopathies have a prodromal window of 5–15 years and slow progression, so cohorts can be moderate in size but must be followed for 12–24 months to observe change [236]. Alzheimer's studies require large groups of participants because changes in sleep and speech are subtle and vary among individuals. Additionally, confirming amyloid or tau levels is necessary to accurately identify the cohort. TDP‐43 disorders present a different challenge: ALS has a short prodromal phase of 1–3 years, progresses rapidly, and requires frequent sampling to detect transitions. A single‐trial design suitable for all three conditions would be too weak for Alzheimer's and too lengthy for ALS. For prodromal Parkinson's, the sample size should be tailored to the specific endpoint [237, 238]. In polysomnography‐confirmed idiopathic REM‐sleep behavior disorder, Alotaibi et al. [239] estimated that detecting a 50% slowing of motor progression over 2 years would require 234 participants per group, whereas a time‐to‐event analysis of motor and cognitive milestones would require 126 participants per group.

Decentralized and hybrid trial designs facilitate real‐world monitoring without frequent site visits. Remote consent, device shipment, and telehealth check‐ins help maintain participant engagement and lower operational demands. Short standardized tasks, such as 3 minutes of orthostatic standing, 30 seconds of finger tapping, and 2 minutes of walking with turns, act as calibration anchors. These tests have test‐retest intraclass correlation coefficients exceeding 0.75 across different device versions [240, 241]. Del Campo et al. [242] found that real‐time adherence monitoring combined with targeted site support increased mean daily actigraphy adherence from 78.1% (SD 12.4%) to 89.4% (SD 3.2%), equating to roughly 18.7–21.5 hours of wear per day. Digital endpoints should be linked to clinical references: day‐by‐day IMU gait composites are validated against MDS‐UPDRS motor scores and dopaminergic imaging, nocturnal HRV and REM measures are validated with polysomnography, and passive computer‐interaction measures are compared to MoCA subscales [243, 244]. Holmes et al. [245] reported correlations with total MoCA (Spearman's *ρ* = 0.48, *p* < 0.001) and cognitive‐domain scores (*ρ* = 0.42–0.54). Cohort diversity directly determines external validity. Enrollment targets should specify at least 25% darker skin tones for PPG‐based optical sensing, at least 40% women to capture sex‐specific autonomic patterns, rural participants, and the older population [246, 247].

### 6.2 Endpoints, Qualification, and Regulatory Pathways

Regulatory qualification requires three levels of evidence: analytical validity, clinical validity, and clinical utility, each with specific quantitative criteria that wearable biomarker studies must pre‐register and report [248].

Analytical validity confirms that the device accurately and reproducibly measures its target signal. Test‐retest reliability should be reported with confidence intervals; ICC values of 0.75–0.90 indicate good reliability, and values > 0.90 indicate excellent reliability [249, 250]. Inter‐device bias and Bland‐Altman limits of agreement should be evaluated against an appropriate reference during expected movement, temperature and placement variations, using endpoint‐specific acceptance criteria. For example, Liang et al. [251] reported mean Oura‐ECG RMSSD biases of 2.50 ms in younger adults and 3.79 ms in older adults.

Clinical validity refers to the biomarker's ability to accurately reflect disease state, severity, or progression. Relevant endpoints should align with clinically meaningful outcomes such as time to diagnosis, progression on the MDS‐UPDRS, fall incidence, or hospitalization [252]. Clinical utility represents the highest level of evidentiary standard. It requires randomized evidence demonstrating that biomarker use leads to measurable improvements in outcomes, such as earlier diagnosis by 3–6 months, a 20%–30% reduction in fall‐related hospitalizations, or quality‐of‐life improvements exceeding the minimally important difference threshold. Claims of utility must be supported by a predefined algorithm change‐control protocol to ensure that model updates do not undermine the validity of the supporting evidence [253].

### 6.3 Integration With EHRs

Validated digital biomarkers deliver clinical value only when integrated into existing clinical workflows. The HL7 FHIR standard facilitates this by providing a structured format for exchanging health data [254]. FHIR uses Observation resources, linked to LOINC codes, and Device resources containing calibration metadata. SMART on FHIR enables OAuth2‐secured app launches within electronic health record systems, while CDS Hooks offers risk summaries in the clinician dashboard [255]. Data governance should comply with HIPAA and GDPR standards. Implementing de‐identification, encryption during storage and transmission, secure on‐device processing enclaves, and auditable access logs helps reduce privacy risks and ensure regulatory compliance [256]. Operational integration should prioritize clinically interpretable summaries over unrestricted, high‐frequency data streams in the EHR. Each digital biomarker must retain its measurement window, device and algorithm version, signal quality, missingness, and provenance, with patient‐generated data clearly distinguished from clinician‐verified information. Dashboards should display longitudinal trends and clinically meaningful deviations, with assigned responsibility for reviewing and escalating abnormalities [257]. Alerts should be severity‐tiered and actionable, while alert frequency, overrides and response times should be monitored after deployment. Multidisciplinary governance and clinician feedback are necessary to remove low‐value notifications and limit alert fatigue [258].

### 6.4 Implementation Science and Global Deployment

Scaled adoption requires a clear implementation strategy, alongside technical validity and regulatory approval. Effective technologies often do not reach patients, particularly in low‐ and middle‐income regions. From an implementation science perspective (e.g., RE‐AIM), reach depends on affordable devices and digital literacy support, while adoption depends on clinician incentives and the extent to which the technology fits into existing workflows [259]. Where connectivity is limited, offline‐first architectures buffer data locally and synchronize opportunistically. On‐device feature extraction reduces transmitted volume relative to raw streaming, making deployment feasible in low‐bandwidth settings [260]. Devices should meet IP67 ingress protection, tolerate high humidity and heat, and provide 5–7 days of battery life. Vendor take‐back for e‐waste should be contractually enforced [261]. National pilots are typically governed through public–private partnerships with outcome‐based contracts and explicit provisions for access, literacy and culturally appropriate design [262]. Scale‐up should proceed in stages, with progression beyond the pilot conditional on prespecified measures of acceptability, adoption, appropriateness, feasibility, fidelity, implementation cost, penetration and sustainability [263]. Researchers should also consider device uptime, data yield, participant retention, clinician workload, cost per active patient, and reach equity. Hybrid effectiveness–implementation designs can assess clinical benefits and delivery strategies simultaneously. This helps differentiate between failures of the technology and failures of implementation [264].

### 6.5 Real‐World Barriers: Adherence, Dropout, Data Loss, and Clinical Responsibility

Adherence is not a fixed property of a device; it depends on factors such as burden, feedback, and disease stage. Protocols that encourage active engagement tend to yield high short‐term adherence, as seen with the Roche PD Mobile App, which reported 96.3% daily compliance over 2 weeks. Nonetheless, this adherence typically declines over time without ongoing prompts. Passive monitoring usually results in lower peak adherence but declines more gradually because it requires less effort. Factors influencing dropout include perceived lack of benefit, device discomfort or visibility, and the need for daily active tasks. Attrition is not random; those who drop out early are more likely to do so, which can bias longitudinal results, suggesting slower disease progression. Therefore, trials should stratify adherence reporting by relevant variables, designate a minimum‐adherence subgroup beforehand, and treat dropout as informative censoring rather than ignoring it [265, 266, 267, 268].

Device dropout includes battery failure, firmware faults, electrode adhesive failure, loss, and damage, leading to device replacements over the course of long‐term deployments that may disrupt data if firmware or calibration differ. Protocols should document device changes, recalibrate after replacements, and include device identity as a covariate in analysis [269]. Without these measures, a firmware update may be mistaken for a change in disease state, introducing spurious drift into longitudinal trajectories. Systems should display data gaps, set a minimum data threshold for risk scores, and avoid low‐confidence scores that may mislead clinicians [270]. Continuous monitoring creates a clinical responsibility that intermittent assessment does not, although the precise legal duty depends on jurisdiction and whether the data have been incorporated into clinical care [271]. Four aspects of the deployment protocol require explicit written agreement. First, the monitoring window: whether alerts are reviewed continuously, during working hours, or asynchronously, and what patients are informed about this. Second, the responsible clinician: a designated role rather than a service. Third, the escalation pathway and response time, including fallback procedures when the primary responder is unavailable. Fourth, the status of the algorithm output: decision support, which maintains clinician responsibility, or autonomous determination, which entails different regulatory and liability considerations [272, 273].

## 7 Toward Trustworthy and Scalable Brain Health Monitoring: An Engineered Ecosystem

Achieving clinical validation is only the beginning; the greater challenge is delivering reliable, scalable, and equitable digital health solutions across diverse real‐world settings. To support widespread deployment, wearable systems must be designed with privacy, fairness, security, and sustainability as core engineering requirements rather than afterthoughts [274, 275].

This section outlines the key technical and ethical constraints, introduces the concept of a Personalized Brain‐Health Operating System as a unifying framework for continuous monitoring and decision support, and discusses the benchmarking, governance, and accountability mechanisms required for long‐term clinical adoption.

### 7.1 Privacy, Security, Fairness, and Sustainability by Design

Moving from standalone wearable devices to a learning health system requires clear governance regarding what data are collected, what inferences can be made, and how the information is used. A key principle is data minimization: once clinically relevant features, such as HRV risk scores from inter‐beat intervals or tremor metrics from accelerometry, have been extracted, retaining the raw physiological waveforms often provides limited additional clinical value and should be avoided where possible [276, 277]. De‐identification alone is insufficient because aggregated activity data can still enable re‐identification at high rates. Therefore, de‐identified aggregates should be retained for no more than 90 days with automatic, tamper‐evident deletion. Differential privacy, with epsilon values around 1–8, should be implemented, with its utility impact reported rather than assumed. Additionally, data releases must pass membership‐inference and linkage‐attack audits, with any vulnerabilities addressed within 30 days [278, 279]. Privacy‐preserving computation should be applied before data leaves the device or the clinical institution [280]. Kaissis et al. [281] recommend combining distributed learning with controls such as secure aggregation, access management and differential privacy. Audits should assess data quality, missingness, calibration and error rates across demographic, clinical and device subgroups, including uncertainty at the intersection of multiple attributes [282]. For example, Yuan et al. [283] identified differences in subgroup sensitivity across machine‐learning models of Alzheimer disease progression.

Additionally, Table 6 outlines the critical engineering requirements for trustworthy brain‐health monitoring, including common failure modes, available evidence, and their implications for the pBH‐OS framework. These considerations provide guidance for designing secure, fair, privacy‐preserving, and clinically reliable wearable health systems.

**TABLE 6:** Engineering requirements for trustworthy brain‐health monitoring: failure modes, evidence and implications for the pBH‐OS.

Requirement	Failure mode addressed	Reported finding	Setting and scale	Implication for pBH‐OS	Citation
Data minimization	Re‐identification from de‐identified activity data	Machine learning re‐identified ∼95% of adults and ∼80% of children from 20‐min aggregated physical‐activity data with demographics; temporal aggregation did not materially reduce accuracy	14,451 individuals, NHANES 2003–06	De‐identification alone is insufficient; raw waveform retention must be minimized, not merely anonymized	[284]
Retention limits	Re‐identification from sparse attributes	99.98% of individuals would be correctly re‐identified in any dataset using 15 demographic attributes	Generative‐model estimation, US population	Justifies the 90‐day retention ceiling and automatic deletion with tamper‐evident logging	[285]
Differential privacy	Privacy budget chosen without utility evidence	Scoping review of differential privacy in health research; most applications remain theoretical or simulated rather than deployed, and ε selection is rarely justified	Systematic scoping review	The ε 1–8 budget must be reported with its measured utility cost, not asserted	[286]
Federated training	Single‐site models fail to generalize	AUC > 0.92 at 24 and 72 h; 16% improvement in average AUC and 38% increase in generalizability versus locally trained models	20 institutions, international	Quantifies the benefit that the pBH‐OS data layer must deliver to justify federated complexity	[287]
Federated training at scale	Rare‐disease cohorts too small at any one site	Federated consortium assembled the largest dataset of its kind and improved delineation of surgically targetable tumor regions relative to a public‐data model	71 sites, 6314 patients, 6 continents	Establishes the feasibility of the multi‐site model hub for TDP‐43 spectrum cohorts	[288]
Fairness—label choice	Proxy outcome encodes structural inequity	At equal risk score, Black patients were considerably sicker; correcting the proxy raised the proportion of Black patients receiving additional care from 17.7% to 46.5%	Commercial algorithm affecting millions of patients	Endpoint definition, not model architecture, is the primary fairness control point	[289]
Fairness—sensor physics	Optical measurement bias by skin pigmentation	Black patients had nearly three times the frequency of occult hypoxemia undetected by pulse oximetry compared with White patients	Two cohorts: University of Michigan and 178 ICUs	Directly grounds the ≥ 25% darker‐skin‐tone enrollment requirement for PPG‐derived endpoints	[290]
Fairness—subgroup reporting	Aggregate accuracy conceals subgroup harm	Consistent underdiagnosis in underserved subgroups, greatest at the intersection of multiple attributes, despite competitive aggregate performance	Three large public chest radiograph datasets	Justifies replacing aggregate AUROC with intersectional subgroup calibration as the reporting standard	[291]
External validation	Vendor‐reported performance not reproduced in service	Proprietary sepsis model achieved AUC 0.63, substantially below developer‐reported performance; sepsis occurred in 7% of hospitalizations	27,697 patients, 38,455 hospitalizations	Post‐market monitoring must re‐estimate performance locally, not inherit the vendor figure	[292]
Regulatory evidence	Clearance without prospective or multi‐site data	Most FDA‐cleared AI devices were evaluated retrospectively; few reported multi‐site evaluation or site‐stratified performance	Analysis of FDA‐cleared AI devices	Sets the evidentiary gap the pBH‐OS acceptance criteria are designed to close	[293]
Jurisdictional divergence	Approval pathways differ between US and EU	Comparative analysis of AI/ML‐based device approvals in the USA and Europe, 2015–2020, showing divergent evidentiary and timing requirements	Regulatory database analysis	Justifies the harmonized change‐control framework proposed in the conclusion	[294]
Trial reporting	AI trial reports omit deployment‐critical detail	CONSORT‐AI extension adds 14 items covering intended use, input handling, human–AI interaction and error analysis	Delphi consensus, international	Defines the reporting floor for any pBH‐OS clinical evaluation	[295]
Early‐stage evaluation	No standard between silent testing and RCT	DECIDE‐AI guideline specifies 17 AI‐specific and 10 generic reporting items for early live clinical evaluation	Delphi consensus, 20 countries	Provides the acceptance criteria for the canary‐cohort stage in section 4.4	[296]
Model documentation	Published models not independently reproducible	MI‐CLAIM checklist defines six required reporting parts spanning study design, data, model, code and reproducibility	Consensus checklist	Specifies the minimum content of the algorithmic accountability report	[297]
Sustainability	Environmental cost excluded from design	Health care accounts for 4.4% of global net greenhouse‐gas emissions	Global assessment, 189 countries	Places device lifecycle emissions inside the engineering specification rather than outside it	[298]
Consent and social license	Data sharing is assumed rather than evidenced	Narrative review: Public support for health‐data sharing is conditional on transparency, governance and absence of commercial exploitation	Narrative review of public and patient attitudes	Grounds the explicit	[299]

### 7.2 The Personalized Brain‐Health Operating System (pBH‐OS): Operationalizing Principles

The pBH‐OS is a conceptual reference architecture, not an implemented platform. All quantitative targets are based on published results for individual components and serve as design goals. The contribution lies in specifying interfaces and measurable requirements between independently developed layers, with Table 7 comparing existing analogs to the gaps. The pBH‐OS organizes the design principles above into a layered, interoperable specification. Contract‐based interfaces between layers allow sensors, models, data stores and clinical interfaces to be updated independently rather than as a monolithic platform.

**TABLE 7:** pBH‐OS layers mapped to their closest deployed or trialed analog, with reported performance and the remaining gap.

pBH‐OS layer	Closest deployed/trialed analog	Reported performance	Maturity	Gap to pBH‐OS specification	Citation
Sensing	Standardized in‐ear EEG, overnight sleep staging	Accuracy 74.1% over five sleep stages; Cohen's κ 0.61 versus full PSG (*n* = 22)	Home‐based validation (recordings took place in participants' homes)	No quality‐index output to downstream layers; no machine‐readable capability schema	[300]
Sensing	Flex‐printed around‐ear EEG, experimenter‐applied at home	10 participants, mean 7.48 h usable data per night; hypnograms scorable from bipolar cEEGrid channels alone (κ = 0.67 vs. frontal‐EEG + EOG)	Home feasibility study	Expert scoring still required; no automated accept/reject contract	[301]
Sensing	Dry user‐generic in‐ear EEG, vigilance state	SVM accuracy 93.2% on previously seen users; 93.3% leave‐one‐user‐out (9 subjects, 35 h)	Prototype, cross‐user validated	Single state variable; no neurodegenerative endpoint; no placement metadata standard	[302]
Sensing	Ring PPG for nocturnal autonomic monitoring	*r* ^2^ 0.996 (nightly mean HR) and 0.980 (HRV); mean bias −0.63 bpm and −1.2 ms versus medical‐grade ECG (*n* = 49)	Consumer device, validated	Proprietary pipeline; per‐segment quality index not exposed to downstream layers	[50]
Sensing	Wrist PPG + accelerometry sleep staging in sleep‐disorder patients	κ 0.62, accuracy 76.4% (244 adults + 48 children, age 3–82); non‐REM parasomnia κ 0.69, REM parasomnia κ 0.55	Clinical population validation	Degrades in exactly the REM‐related conditions most relevant to prodromal synucleinopathy	[206]
Sensing (multimodal body network)	Wrist/ankle multimodal sensing for seizure detection	94 patients (median age 9.9 yr); 548 seizures analyzed; 11,066 h of sensor data; nine seizure types	Epilepsy monitoring unit benchmark	Single‐site data collection; no contract‐based interface between sensing and compute layers	[208]
Near‐sensor compute	On‐device ECG arrhythmia classification, compact transformer	4.28 ms latency; 0.09 mJ per inference; 98.97% accuracy; 98.36% under motion‐artifact augmentation	Benchmark‐validated on MCU	Single task; no calibrated uncertainty output; no neurodegenerative endpoint	[149]
Near‐sensor compute	Multimodal EEG + PPG + accelerometer fusion on ultra‐low‐power MCU	0.11 mJ per inference; 19.25 mW mean power; 64.28% sample sensitivity, 95% of events, 0.21 false positives/h	Clinical dataset validation	Seizure endpoint only; fusion fixed at design time, not reconfigurable by contract	[151]
Near‐sensor compute	Smartphone‐resident five‐stage sleep staging	2.8 ms per 30 s epoch; ∼100 kB memory; 48.95 MFLOPs; accuracy 83.3%, Cohen κ 0.77, subject‐independent across three datasets	Multi‐dataset validation	Demonstrates the edge–hub split is feasible but carries no provenance or missingness mask	[156]
Near‐sensor compute	Compressed multimodal freezing‐of‐gait model on edge TPU	75 ms per window on Coral, 211 ms on Raspberry Pi 4, < 350 ms end‐to‐end; 6.5 MB → 2.9 MB (−54.2%); accuracy 92.5%, AUC 0.91 across four datasets	Cross‐dataset validation	No calibrated abstention or uncertainty output	[159]
Near‐sensor compute (analog front end)	Ultra‐low‐power sEMG acquisition front end	Average current reduced 92.72% versus commercial devices; battery life 9.057× longer; signal correlation up to 99.5%	Hardware validated against commercial reference	Acquisition only; no on‐device inference or quality gating integrated	[303]
Foundation model hub	Multimodal sleep foundation model (SleepFM)	585,000+ *h* from ∼65,000 participants; 130 conditions at C‐index ≥ 0.75; dementia C‐index 0.85; sleep‐staging F1 0.70–0.78; transfers to unseen SHHS cohort	Research release, externally validated	Clinical PSG input, not wearable; no versioned clinical release with subgroup and energy reporting; no change‐control plan	[234]
Closed‐loop therapeutics	Chronic adaptive deep brain stimulation versus conventional DBS	Blinded randomized crossover trial, 4 patients (6 hemispheres); stimulation‐entrained gamma oscillations optimal state marker in all patients	Randomized feasibility trial; device regulatory‐cleared.	Driven by implanted local field potentials only; not integrated with passive wearable monitoring; no formal policy verification	[304]

The Sensing Layer establishes a standardized framework for wearable devices by defining key characteristics such as sampling frequency, dynamic range, noise levels, signal quality metrics, and power consumption in a machine‐readable format. Device‐specific drivers translate these outputs into unified data formats with consistent units, timestamps, and sensor‐placement information. To ensure data reliability, on‐device quality assessment and filtering mechanisms screen incoming signals, allowing only high‐quality data to be passed to higher system layers for further analysis [305]. The Near‐Sensor computation layer handles compression, denoising, and classification within strict energy constraints. It utilizes quantized temporal convolutional networks, masked autoencoders, and compact transformers converted to fixed‐point executables. Its goals include generating freezing‐of‐gait alerts within one second, autonomic prompts within a few seconds, and providing a calibrated uncertainty estimate for each prediction. Raw biosignals remain on‐device unless the user explicitly allows export [306, 307].

The Foundation Model Hub offers pre‐trained encoders for EEG, IMU, PPG/ECG, EOG, and speech, trained through self‐supervised learning on multi‐site data. Each release includes versioned weights, a device calibration table, and a performance report with subgroup results, energy, and latency profiles. Task‐specific heads support diagnosis, progression tracking, event detection, and signal quality assessment, and can be redirected to new objectives without full retraining [308]. Thapa et al. [234] pretrained SleepFM on more than 585,000 hours of multimodal recordings from approximately 65,000 participants; the model integrated EEG, EOG, ECG and other physiological signals, supported task‐specific heads and predicted dementia with a C‐index of 0.85. Domain adapters narrow the cross‐device AUROC gap. A change control plan governs post‐release updates, verification.

The Digital Twin Layer fits personalized generative state‐space models to longitudinal streams and clinical anchors, with latent states representing motor timing, autonomic regulation, sleep architecture and cognitive control, constrained by neurobiological priors [309]. The twin forecasts trajectories with uncertainty bounds and answers counterfactual queries such as advancing an evening dose by 45 minutes. Person‐specific baseline and drift estimates allow alerts to flag deviation from an individual norm rather than a population average, reducing false positives at equal sensitivity [310]. The Closed‐Loop and Policy Layers operate under predefined, verifiable rules to ensure safety, privacy, and accountability. Sensitive data, such as raw EEG recordings, remain on the device unless explicit permission is granted for sharing. Clinical alerts and decision thresholds must adhere to approved fairness and equity standards, and any neuromodulation or intervention must remain within clinician‐defined safety limits. Human oversight is maintained through mandatory review and override mechanisms. In addition, safety policies are continuously validated using anonymized datasets, and simulation environments enable new algorithms to be tested on synthetic or replayed data before real‐world deployment [311]. The seven‐layer structure of the Personalized Brain‐Health Operating System (pBH‐OS) is shown in Figure 7, implementing principles through contract‐based interfaces.

**FIGURE 7:**
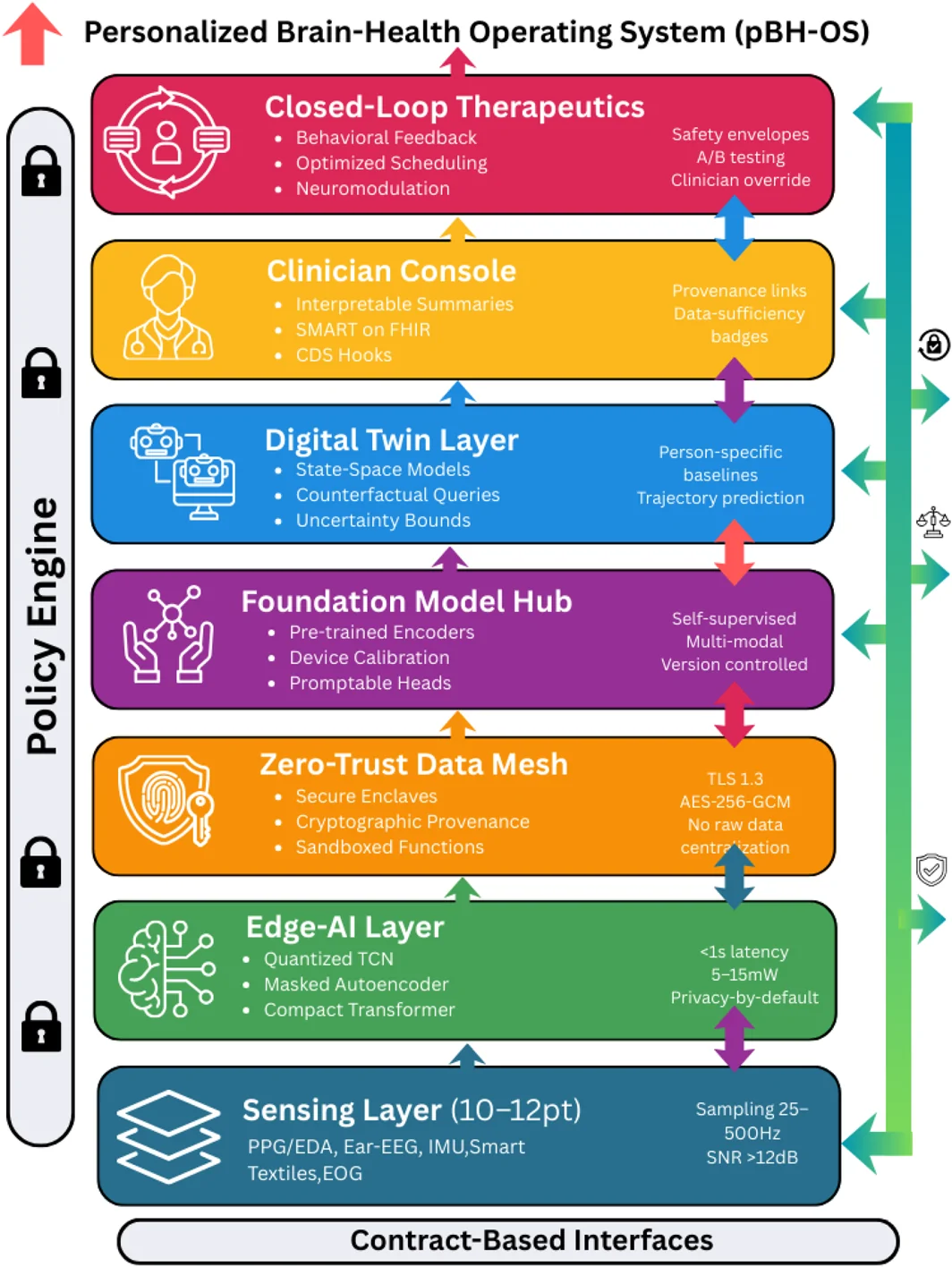
The Personalized Brain‐Health Operating System (pBH‐OS) layered architecture. Seven‐layer stack from sensing to closed‐loop therapeutics, implementing privacy‐by‐design, federated learning, and fairness‐aware deployment through contract‐based interfaces with quantified design targets.

### 7.3 Validation Infrastructure: Benchmarks, Accountability, and Governance

Progress stalls when every group evaluates proprietary data with custom metrics. The field needs open benchmarks that explicitly test generalization across sites, devices and time [61]. Benchmark tasks should be clinically specific, including onset risk prediction within 12–24 months, estimating individual progression rates, and detecting transient decline [312]. Generalization should be tested explicitly across location, device, and time to assess regional, device‐specific, and temporal variations. Implementing strict controls helps prevent data leakage by segregating households, weeks, and patients across datasets and using actual timestamps for partitioning. Clearly defined and published labels are essential. Confirm REM atonia loss with two independent scorers via polysomnography within 3 days. Labels for motor deterioration should be determined through a blinded review of IMU data and clinical notes related to dose adjustments [313, 314].

Leaderboards must require submissions to include subgroup performance, calibration, energy per inference, end‐to‐end latency on reference hardware, memory footprint, and model size [315]. Composite performance metrics should balance accuracy, calibration, fairness, and efficiency, rather than optimizing only predictive performance. To support transparency, dataset registries should document key characteristics, including data modality, cohort size, sampling frequency, label density, accessibility, and environmental context.

In addition, each model release should be accompanied by an Algorithmic Accountability Report detailing its intended use, training data, evaluation cohorts, subgroup performance, calibration, known limitations, and version history. Independent audits and compliance with recognized quality, safety, and privacy standards (e.g., ISO 13485, ISO 14971, IEC 62304, ISO 27001, and ISO 27701) are essential to ensure trustworthiness and regulatory readiness [316, 317].

Consent should be explicit and comprehensible. Participants must quickly understand what data is collected, why it is collected, how long it will be kept, and with whom it is shared, with the option to pause and resume without uninstalling [318]. Clinicians need dashboards that show whether there is enough data, include confidence intervals, and display the latest model updates. This helps ensure scores are not accepted blindly when there is not enough evidence [280, 319]. This requirement pertains to the interface aspect of the abstention policy described in Section 6.5. It tackles risks associated with dual‐use technology, such as the potential to repurpose tremor sensors for surveillance, to use voice data features for identity profiling, and to raise human rights issues related to the scope of features and coding. To promote responsible deployment, it is essential to implement contractual restrictions and conduct comprehensive internal reviews to assess new use cases for their benefits, necessity, and proportionality prior to enabling production [320, 321].

The governing principle is a contract with four terms: measurement consistent with sensor physics and human physiology, models that learn without leaking information, decisions that can be explained, and interventions designed to help patients rather than merely to intervene [322]. The pBH‐OS outlines how the contract could be implemented, with open benchmarks used to determine compliance. This framing treats privacy, fairness, governance, and sustainability as essential engineering requirements, each with measurable targets and transparent reporting.

## 8 Conclusion

Wearable technologies have the potential to detect neurodegenerative diseases during their prodromal stages, but clinical success requires rigorous integration of biological understanding, sensor engineering, signal processing, machine learning, and clinical validation. No single component can compensate for weaknesses elsewhere in the pipeline.

Several principles are essential. Wearable biomarkers must be biologically grounded, linking pathology and neural dysfunction to measurable physiological signals. Scalable deployment depends on edge AI, federated learning, and efficient on‐device processing. Fairness, privacy, security, and sustainability should be treated as core design requirements rather than afterthoughts. Furthermore, the heterogeneity of synucleinopathies, Alzheimer's disease, and TDP‐43 disorders demands disease‐specific sensing and analytical strategies.

Key challenges remain, including validating multimodal biomarkers against clinical and pathological ground truth, improving cross‐site and cross‐device generalization, developing foundation models for physiological data, and establishing harmonized regulatory frameworks for adaptive AI. Ultimately, the clinical value of wearable AI will depend on systems that are accurate, transparent, fair, and trustworthy throughout the process, from data acquisition to clinical decision‐making.

## Author Contributions

**Muhammad Hasan Masrur:** conceptualization, writing – original draft. **Bangul Khan:** conceptualization, writing – original draft, writing – review and editing. **Rana Talha Khalid:** writing – original draft. **Sumaira:** writing – original draft. **Saad Abdullah:** conceptualization, writing – original draft, writing – review and editing, visualization, supervision.

## Conflicts of Interest

The authors declare no conflicts of interest.

## Data Availability

Data sharing not applicable to this article as no datasets were generated or analyzed during the current study.
